# Surrogate-driven respiratory motion model for projection-resolved motion estimation and motion compensated cone-beam CT reconstruction from unsorted projection data

**DOI:** 10.1088/1361-6560/ad1546

**Published:** 2024-01-12

**Authors:** Yuliang Huang, Kris Thielemans, Gareth Price, Jamie R McClelland

**Affiliations:** 1 Centre for Medical Image Computing, University College London, London, United Kingdom; 2 Wellcome/EPSRC Centre for Interventional and Surgical Sciences, University College London, London, United Kingdom; 3 Institute of Nuclear Medicine, University College London, London, United Kingdom; 4 Christie NHS Foundation Trust, Manchester, United Kingdom

**Keywords:** dynamic CBCT, motion model, motion compensation

## Abstract

*Objective.* As the most common solution to motion artefact for cone-beam CT (CBCT) in radiotherapy, 4DCBCT suffers from long acquisition time and phase sorting error. This issue could be addressed if the motion at each projection could be known, which is a severely ill-posed problem. This study aims to obtain the motion at each time point and motion-free image simultaneously from unsorted projection data of a standard 3DCBCT scan. *Approach.* Respiration surrogate signals were extracted by the Intensity Analysis method. A general framework was then deployed to fit a surrogate-driven motion model that characterized the relation between the motion and surrogate signals at each time point. Motion model fitting and motion compensated reconstruction were alternatively and iteratively performed. Stochastic subset gradient based method was used to significantly reduce the computation time. The performance of our method was comprehensively evaluated through digital phantom simulation and also validated on clinical scans from six patients. *Results.* For digital phantom experiments, motion models fitted with ground-truth or extracted surrogate signals both achieved a much lower motion estimation error and higher image quality, compared with non motion-compensated results.For the public SPARE Challenge datasets, more clear lung tissues and less blurry diaphragm could be seen in the motion compensated reconstruction, comparable to the benchmark 4DCBCT images but with a higher temporal resolution. Similar results were observed for two real clinical 3DCBCT scans. *Significance.* The motion compensated reconstructions and motion models produced by our method will have direct clinical benefit by providing more accurate estimates of the delivered dose and ultimately facilitating more accurate radiotherapy treatments for lung cancer patients.

## 1. Introduction

As one of the major therapies for lung cancer of all stages, over half of all patients receive radiotherapy (Brown *et al* 2019). Modern intensity modulated radiotherapy can produce dose distributions that are highly conformal to the shape of tumor so as to deliver high radiation dose to the tumor while sparing the surrounding normal tissues (Pirzkall *et al* 2000). However, the patients anatomy can change during the course of treatment (Cole *et al* 2018) which can lead to the tumor receiving less dose and/or the normal tissues receiving more dose than planned (den Otter *et al* 2020).

On-board cone-beam CT (CBCT), which is integrated on most clinical linear accelerators nowadays (De Los Santos *et al* 2013), has been widely investigated for Adaptive Radiotherapy (ART) to account for patients inter-fraction anatomical changes (Cole *et al* 2018). However, artifacts due to respiration motion usually degrade the quality of the CBCT images (Sweeney *et al* 2012). In addition, standard 3DCBCT provides no information on the tumor motion, which can change between between sessions of daily radiotherapy (Dhont *et al* 2018).

To inform ART with motion-of-the-day, the scan duration can be increased 2–4 times so as to acquire more projections that can then be sorted into multiple respiratory phases (normally 6–10 phases) to obtain 4DCBCT (Sonke *et al* 2005), i.e. a series of CBCT images reconstructed out of binned projections that belong to each respiratory phase. Nevertheless, 4DCBCT suffers from severe streak artifacts due to the uneven angular distribution of projections within each phase (Leng *et al* 2008), and breath-to-breath variability and sorting errors can lead to residual blurring. Moreover, the longer acquisition time of 4DCBCT is undesirable in clinical practice and associated with more imaging dose to patients (Thengumpallil *et al* 2016).

Various methods can be utilized to improve the quality of 4DCBCT. One type of method exploits compressed sensing theory for sparse-view reconstruction, e.g. using different kinds of edge-preserving total variation (Jia *et al* 2010) or prior-image-constrained-compressed-sensing (Chen *et al* 2008). Another type of method utilizes deep learning techniques to postprocess the CBCT images reconstructed with under-sampled data to achieve similar image quality as CBCT images reconstructed with full data (Jiang *et al* 2019). However, these methods reconstruct the CBCT image for each phase separately and do not take advantage of the temporal redundancy of the CBCT data. Other studies (Mory *et al* 2014, 2016, Zhi *et al* 2021) applied temporal regularization as well as spatial regularization on 4DCBCT reconstruction. Nevertheless, 4DCBCT assumes periodic breathing, which is not always a valid assumption for lung cancer patients (Nøttrup *et al* 2007).

In comparison to 4DCBCT, the motion compensated approach estimates the motion that occurred during the CBCT acquisition and uses the motion estimation to reconstruct a single ‘static’ 3D image through motion compensated versions of Feldkamp–Davis–Kress (FDK) (Rit *et al* 2009) or iterative reconstruction (Chee *et al* 2019) algorithms. Since all the projections are used in reconstructing the image, the image quality should not be influenced by under-sampling effects, but the difficulty of this approach lies in accurately estimating the motion. Some methods estimated the motion from the planning 4DCT scan (Rit *et al* 2009), but changes to the motion and/or anatomy between the 4DCT and CBCT scan could limit the accuracy of such approaches. Other methods used 4DCBCT to estimate the motion model (Guo *et al* 2019) or iteratively registered and reconstruct 4DCBCT in a multi-resolution approach (Wang and Gu 2013), where image registration between different phases of 4DCBCT is required but the poor image quality could limit the accuracy of these approaches. Some studies used deep-learning based approaches to improve the quality of the initial 4DCBCT images and the subsequent image registration step (Yang *et al* 2022, Zhang *et al* 2023), but these methods could not account for breath-to-breath variation.

Alternatively, surrogate driven motion models (McClelland *et al* 2013) can estimate the motion at each timepoint, with the capability to capture breath-to-breath variability. In this approach, the motion is parameterized by one or more respiratory surrogate signals which can be acquired from external devices, such as marker(s) on the skin surface (Hurwitz *et al* 2014, Dong *et al* 2023), or derived directly from the projection data using the Amsterdam shroud method (Zijp *et al* 2004) or similar techniques (Kavanagh *et al* 2009). The common approach for fitting a surrogate-driven motion model consists of two separate steps, i.e. first performing image registration to get 3D motion fields and then fitting the relation between the 3D motion fields and the corresponding surrogate signals (McClelland *et al* 2013). However, to obtain 3D motion field requires dynamic 3D images, which would most likely be obtained by pre-existing phase-sorted 4D images and thus lose information of breath-to-breath variability. In recent years a general motion modelling framework has been proposed that unifies the image registration and motion model fitting steps into a single optimization process, enabling the method to be applied on unreconstructed ‘raw’ data such as CBCT projections (McClelland *et al* 2017). Moreover, motion compensated reconstruction could also be integrated into this method so no prior image is required. This framework has been optimized and extensively evaluated for multi-slice MRI data from a MR-Linac (Tran 2022).

This paper adapted the framework above so it could be applied to CBCT projection data with several technical developments, which include incorporating forward- and back-projection operators (from openRTK) into the framework, implementing a new similarity measure (LNCC), implementing a motion-compensated FDK reconstruction that utilizes the projection-specific motion estimates provided by the motion model, and implementing a stochastic gradient descent optimization scheme which greatly improves the computation time. Additionally, a method for extracting the surrogate signal from the projection data was implemented and utilized. The contributions of this paper also include thorough quantitative and qualitative evaluation using simulated data from a computer phantom. More importantly, the feasibility of this method is demonstrated on real patient data. Using our method, dynamic images that showed the respiration motion of lung cancer patients can be generated from nothing more than unsorted CBCT projection in a standard 3D CBCT scan. An earlier version of this paper was presented at the IEEE international Symposium of Biomedical Imaging and was published in its proceedings (Huang *et al* 2023).

## 2. Material and methods

### 2.1 The general motion modelling framework

Full details of the general framework can be found in (McClelland *et al* 2017), but for the sake of clarity, a brief description of the framework, and how it has been adapted for CBCT projection data, will be given below.

Given a set of 2D CBCT projection images (**P**_**t**_), the goal of this study is to obtain a motion-free CBCT image (**I**_**0**_) and a time series of deformation vector fields (DVFs) **D**_**t**_ that can warp the reference state image **I**_**0**_ to the CBCT image (**I**_**t**_) at the moment when each projection was acquired using

\begin{eqnarray*}{{\bf{I}}}_{{\bf{t}}}=T({{\bf{I}}}_{{\bf{0}}},{{\bf{D}}}_{{\bf{t}}}),\end{eqnarray*}

where *T* is a function that resamples **I**_**0**_ according to the spatial transform determined by **D**_**t**_ at time-point *t*.

This study used a B-spline free-form deformation (FFD) transformation model:

\begin{eqnarray*}{{\bf{D}}}_{{\bf{t}}}=\phi ({{\bf{M}}}_{{\bf{t}}}),\end{eqnarray*}

where *ϕ* is a function based on cubic B-splines, that takes the control point grid displacements that define the FFD, **M**_**t**_, as input and returns the voxel-wise DVF, **D**_**t**_. The surrogate-driven respiration correspondence model can then be represented as follows:

\begin{eqnarray*}{{\bf{M}}}_{{\bf{t}}}={{\bf{S}}}_{{\bf{t}}}\cdot {\bf{C}}={{\mathrm{\Sigma }}}_{i=1}^{{N}_{s}}{S}_{{it}}\cdot {{\bf{C}}}_{{\bf{i}}}\end{eqnarray*}

in which *N*_*s*_ is the number of surrogate signals, *S*_*it*_ is the *i*th surrogate signal at time-point *t* and **C**_**i**_ is the *i*th component of correspondence model parameters. At least two surrogate signals are required to model both intra- and inter-cycle variability (McClelland *et al* 2013). More signals can be used, but this increases the number of model parameters and the danger of overfitting the data. Furthermore, there is some evidence in the literature that just two signals can approximate the motion well over a few minutes (Liu *et al* 2012, Manber *et al* 2016, Tran *et al* 2020), so two signals were used in this study.

The motion model parameters **C** can be determined by minimizing the loss function below:

\begin{eqnarray*}f=-{{\mathrm{\Sigma }}}_{t=1}^{{N}_{t}}L({{\bf{P}}}_{{\bf{t}}},{{\bf{P}}}_{{\bf{t}}}^{\prime} )\end{eqnarray*}

\begin{eqnarray*}{{\bf{P}}}_{{\bf{t}}}^{\prime} ={{\bf{A}}}_{{\bf{t}}}\cdot {{\bf{I}}}_{{\bf{t}}}\end{eqnarray*}

where *L* refers to the localized normalized cross correlation, ${{\bf{P}}}_{{\bf{t}}}^{\prime} $ and **P**_**t**_ are the estimated and measured projection images at time *t* respectively, and **A**_**t**_ is the acquisition matrix for CBCT forward projection. Voxels outside the reconstruction field-of-view (FOV) will be set to null value so that **A**_**t**_ will ignore those voxels and prevent them from interfering with loss function calculation.

Combining equations (1)–(5) the gradient of the loss function with respect to the motion model parameters is:

\begin{eqnarray*}\begin{array}{l}\displaystyle \frac{\partial f}{\partial {{\bf{C}}}_{{\bf{i}}}}={{\mathrm{\Sigma }}}_{t}\displaystyle \frac{\partial {{\bf{M}}}_{{\bf{t}}}}{\partial {{\bf{C}}}_{{\bf{i}}}}\cdot \displaystyle \frac{\partial {{\bf{D}}}_{{\bf{t}}}}{\partial {{\bf{M}}}_{{\bf{t}}}}\cdot \displaystyle \frac{\partial {{\bf{I}}}_{{\bf{t}}}}{\partial {{\bf{D}}}_{{\bf{t}}}}\cdot \displaystyle \frac{\partial {{\bf{P}}}_{{\bf{t}}}^{\prime} }{\partial {{\bf{I}}}_{{\bf{t}}}}\cdot \displaystyle \frac{\partial f}{\partial {{\bf{P}}}_{{\bf{t}}}^{\prime} }\\ \,=-{{\mathrm{\Sigma }}}_{t}{S}_{{it}}\cdot \displaystyle \frac{\partial {{\bf{D}}}_{{\bf{t}}}}{\partial {{\bf{M}}}_{{\bf{t}}}}\cdot \displaystyle \frac{\partial {{\bf{I}}}_{{\bf{t}}}}{\partial {{\bf{D}}}_{{\bf{t}}}}\cdot {{\bf{A}}}_{t}^{* }\cdot \displaystyle \frac{\partial L({{\bf{P}}}_{{\bf{t}}},{{\bf{P}}}_{{\bf{t}}}^{\prime} )}{\partial {{\bf{P}}}_{{\bf{t}}}^{\prime} }\end{array}\end{eqnarray*}

where *i* = 1,…,*N*_*s*_, and ${{\bf{A}}}_{t}^{* }$ is the adjoint matrix of **A**_**t**_. The gradient can be calculated over all the projections or a subset of projections. For the sake of computation efficiency, the model fitting used evenly-spaced subsets, with just one-tenth of the projections in each, and stochastic gradient descent, reducing computation time by a factor of ∼10 per update step.

In the proposed method, **I**_**0**_ is initially reconstructed using the standard FDK algorithm (Feldkamp *et al* 1984). This is used to fit the motion model parameters, **C**. However, the accuracy of this initial fit may be limited due to the motion artifacts in **I**_**0**_. Therefore, **I**_**0**_ and **C** are alternatingly updated by performing a motion compensated FDK reconstruction (Rit *et al* 2009) and fitting the motion model parameters as described above.

The proposed method was implemented by adapting our open-source software SuPReMo (https://github.com/UCL/SuPReMo). We used openRTK (Rit *et al* 2014) for forward and back projection but implemented the motion compensated reconstruction by ourselves by warping each back projection volume. A dockerized implementation will be made available after reasonable request to the authors.

The hyperparameters used for this study are: control point grid spacing of 8 voxels, maximum number of motion compensated reconstructions per level was 6, maximum number of model fitting iterations was 100. A multi-resolution approach was adopted, with **P**_**t**_, **C**, and **I**_**0**_ being resampled at each resolution level. Two resolution levels were used, i.e. 1/4 and 1/2 of the original resolution, as we found that fitting the motion model at the original resolution level greatly increased the runtime for little or no improvement to the model accuracy.

### 2.2 CBCT acquisition data

#### 2.2.1 Digital phantom simulation

**Figure 1.**
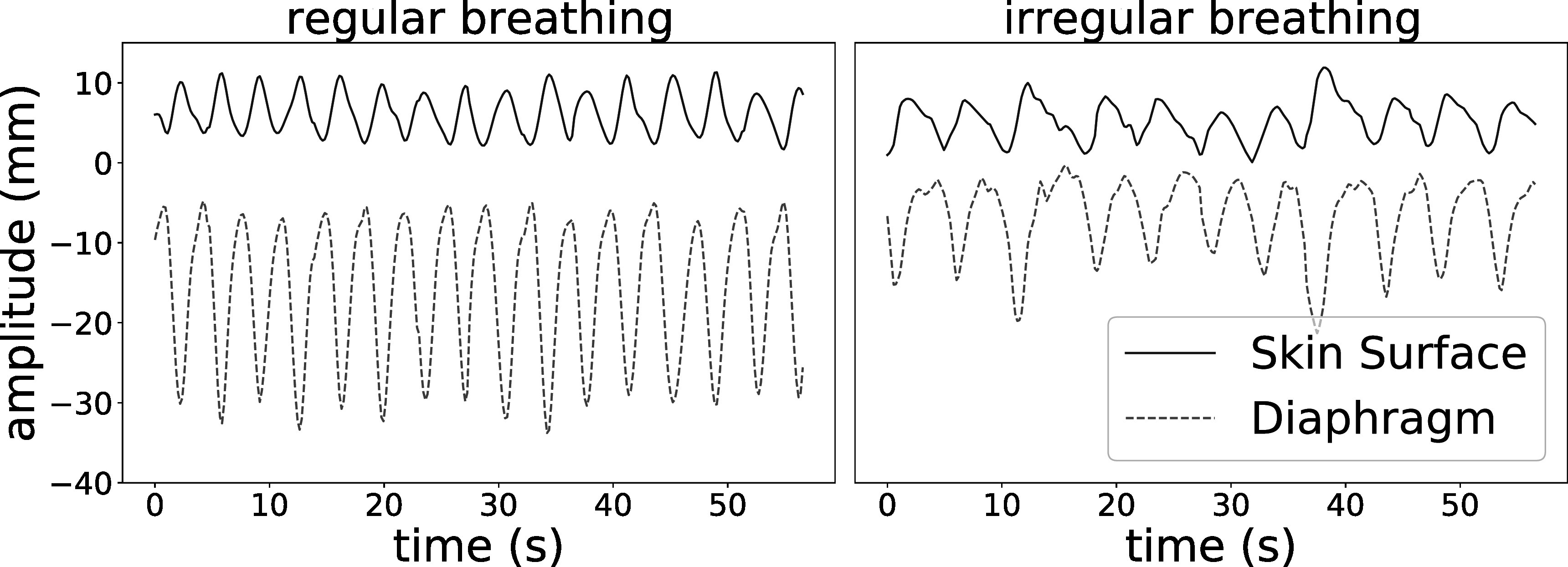
The input breathing traces for XCAT simulation that represent regular breathing (a) and irregular breathing (b).

The XCAT software (Segars *et al* 2010) was used to generate a ground truth reference state image and sequence of DVFs from breathing traces that represent the motion of diaphragm in SI direction and motion of chest surface along AP direction. This study used two sets of real breathing traces, as shown in figure 1, which were measured from cine sagittal MR slices.

The first set of breathing traces showed regular respiration and the other one exhibited a more irregular pattern including hysteresis and inter-cycle variation. More specifically, the two traces are in-phase with each other in the regular simulation, while out-of-phase with each other in irregular simulation, the latter of which also has more variable magnitudes among different breathing cycles.

**Figure 2.**
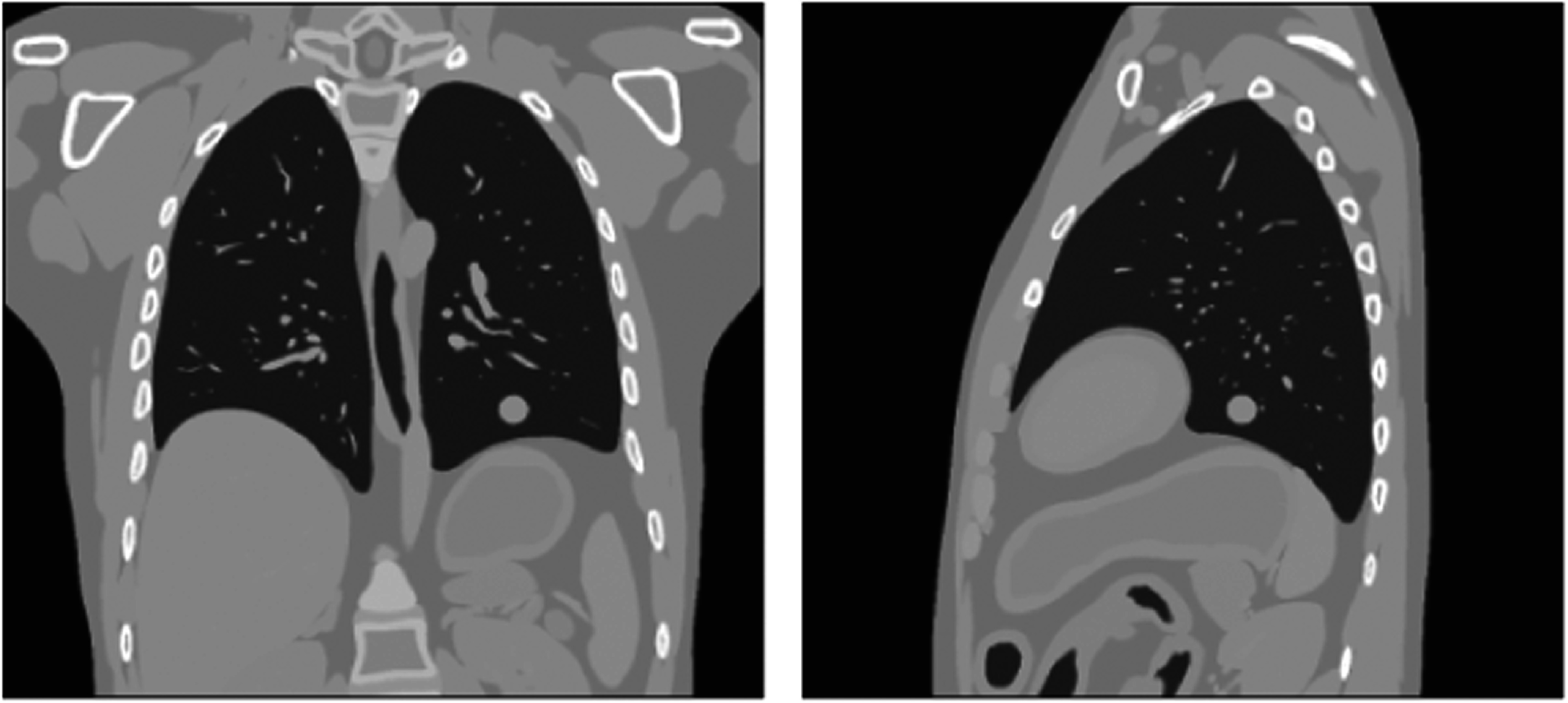
Ground-truth reference state image for the 4DXCAT simulation of irregular breathing.

For both simulations, the reference state image (size: 375 × 375 × 343, resolution: 1 mm × 1 mm × 1 mm) was created representing the time average position of the anatomy over the acquisition. A spherical tumor with radius of 15 mm was added to the reference state images on the lower part of left lung, as shown in figure 2.

The DVFs from the XCAT simulation can cause different structures/organs to move through each other. The CID-X software (Eiben *et al* 2020) was used to post-process the outputs of the XCAT to prevent this happening, and give consistent and invertible DVFs that still preserve the sliding motion between the lungs and the chest wall. These post-processed DVFs provide the ground truth motion, ${{\bf{D}}}_{{\bf{t}}}^{{\bf{gt}}}$, for each time point *t*, and were used to warp the reference state image to produce the dynamic image for each time point. They were also used to warp a mask of the tumor to produce ground truth tumor masks for each time point, ${{\bf{Mask}}}_{{\bf{t}}}^{{\bf{gt}}}$.

Projection images were generated from the dynamic images with OpenRTK (Rit *et al* 2014) using the geometry of a real CBCT scan on an Elekta Synergy (Elekta AB, Stockholm, Sweden) system (scan angle: 360°, source-to-isocenter distance (SID): 1000 mm, source-to-detector distance (SDD): 1536 mm). 310 projections were generated per scan to simulate a one-minute scan at acquisition rate of 5.4 fps. Resolution and dimensional size of the projection images are 0.8 mm × 0.8 mm and 512 × 512 respectively.

#### 2.2.2 Patient data

The approach was verified in 2 real-world patient datasets:

(i) SPARE Challenge dataset (Shieh *et al* 2019). The SPARE challenge dataset includes data from 10 patients, but 6 of these suffer from heavily truncated data (i.e. parts of their anatomy are missing from the reconstructed images due to the limited field of view) and cannot be used in this study. The CBCT images were acquired with a scan angle of 360° on a Varian Trilogy system with SID of 1000 mm and SDD of 1500 mm, with an offset detector to enlarge the field-of-view (FOV) to 450 mm × 450 mm × 220 mm. Dimensions and pixel size of the projection images were 1024 × 768 and 0.388 mm × 0.388 mm respectively. The datasets consist of 680 projections each, equivalent to a standard 1 min 3DCBCT scan, although they have actually been subsampled from longer scans (∼8 min) 4DCBCT scans.
(ii) ROSS-LC clinical trial (Price *et al* 2018). We have also demonstrated our method on true (i.e. not subsampled) clinical 3DCBCT scans from two patients from the ROSS-LC clinical trial (REC ref. 14/NW/0037). The data were acquired using an Elekta XVI (Elekta AB, Stockholm, Sweden) system under standard 3D CBCT settings, i.e. ∼600 projections during a 2 min scan over a full rotation. The SID and SDD are 1000 mm and 1536 mm respectively. Resolution and dimensional size of the projection images are 0.8 mm × 0.8 mm and 504 × 504 respectively. FOVs of the two patients were 410 mm × 410 mm × 264 mm and 410 mm × 410 mm × 168 mm respectively.

## 3. Experiments

### 3.1 Surrogate signal extraction

As external breathing were not available for the clinical datasets, the Intensity Analysis (IA) method (Kavanagh *et al* 2009) was used to extract the surrogate signals directly from the CBCT projection data. Briefly, The IA method calculates the sum of the pixel intensities for each projection, and splits the 1D signal obtained from this into low-frequency and high-frequency components. The low-frequency part reflects slow gantry rotation while the high-frequency part is related to more frequent respiration motion which is used as the surrogate signal. As each model requires two surrogate signals as inputs, the temporal gradient of the IA signal was used as the second surrogate signal when fitting the models. The temporal gradient is used to present breathing rate, in accordance with 5D lung motion model (Low *et al* 2005) that has been supported by many studies (Zhao *et al* 2009, Liu *et al* 2015, Chee *et al* 2019).

To make better use of the simulation dataset for evaluating the impact of surrogate signals, the input breathing traces to the XCAT simulation were also used as another set of surrogate signals to fit another motion model. These should provide the best possible surrogate signals, as they were used to drive the XCAT simulations, but comparable signals are not available for real data. Comparison between different types of signals can reveal the impact of using non-perfect signals. All the surrogate signals were normalized to have mean of 0 and standard deviation of 1.

### 3.2 Evaluation

For simulation data, the performance of the motion model was assessed by the following metrics:

(i) E_D_: The L2-norm of the difference between the ground-truth (${{\bf{D}}}_{{\bf{t}}}^{{\bf{gt}}}$) and estimated (${{\bf{D}}}_{{\bf{t}}}^{{\bf{est}}}$) DVFs averaged over all the time-points *N*_*t*_ and a Volume-of-Interest (VOI) defined as the human body within the reconstruction FOV:
  \begin{eqnarray*}{{\mathrm{E}}}_{D}=\displaystyle \frac{1}{{N}_{t}\times | \mathrm{VOI}| }{{\mathrm{\Sigma }}}_{t=1}^{{N}_{t}}{{\mathrm{\Sigma }}}_{{\bf{x}}\in \mathrm{VOI}}| {{\bf{D}}}_{{\bf{t}}}^{{\bf{est}}}({\bf{x}})-{{\bf{D}}}_{{\bf{t}}}^{{\bf{gt}}}({\bf{x}})| \end{eqnarray*}
(ii) DSC: DICE similarity coefficient between the estimated tumor masks (${{\bf{Mask}}}_{{\bf{t}}}^{{\bf{est}}}$) and the ground-truth tumor masks (${{\bf{Mask}}}_{{\bf{t}}}^{{\bf{gt}}}$) averaged over *n*_*t*_ time-points:
  \begin{eqnarray*}\mathrm{DSC}=\displaystyle \frac{1}{{n}_{t}}{{\mathrm{\Sigma }}}_{t=1}^{{N}_{t}}\displaystyle \frac{2\times | {{\bf{Mask}}}_{{\bf{t}}}^{{\bf{est}}}\cap {{\bf{Mask}}}_{{\bf{t}}}^{{\bf{gt}}}| }{| {{\bf{Mask}}}_{{\bf{t}}}^{{\bf{est}}}| +| {{\bf{Mask}}}_{{\bf{t}}}^{{\bf{gt}}}| }\end{eqnarray*}
(iii) E_center_: The Euclidean distance between estimated ${{\bf{c}}}_{t}^{{est}}$ and ground-truth ${{\bf{c}}}_{t}^{{gt}}$ tumor centroid positions averaged over *n*_*t*_ time-points:
  \begin{eqnarray*}{{\mathrm{E}}}_{\mathrm{center}}=\frac{1}{{n}_{t}}{{\mathrm{\Sigma }}}_{t=1}^{{N}_{t}}| | {{\bf{c}}}_{{\bf{t}}}^{{\bf{est}}}-{{\bf{c}}}_{{\bf{t}}}^{{\bf{gt}}}| {| }_{2}\end{eqnarray*}
(iv) NRMSE: normalized root-mean-square-error (normalized to the maximum pixel value of ground-truth):
  \begin{eqnarray*}\mathrm{NRMSE}=\displaystyle \frac{1}{\mathrm{Max}({{\bf{I}}}_{{\bf{0}}}^{{\bf{gt}}})}\cdot \sqrt{\displaystyle \frac{1}{| {VOI}| }\cdot | | {{\bf{I}}}_{{\bf{0}}}^{{\bf{est}}}-{{\bf{I}}}_{{\bf{0}}}^{{\bf{gt}}}| {| }^{2}}\end{eqnarray*}
  where *I*_0_^*gt*^ is the motion compensated FDK reconstruction using ground-truth DVFs and *I*_0_^*est*^ is the motion compensated FDK reconstruction using model estimated DVFs.
(v) SSIM: structural similarity index between *I*_0_^*gt*^ and *I*_0_^*est*^.

For real patient data, as ground-truth DVFs were not available, visual inspection was used to evaluate the quality of the reconstructed image. Visualising the results for both the simulated and real datasets can be found in the supplementary material.

### 3.3 Comparing scenarios

Three scenarios were compared using the metrics above:

**S**_**uncorr**_: Uncorrected results, i.e. not involving motion compensation. The tumor masks at all time-points are the same as the mask on the average position image. DVFs are zero over space and time. Reconstruction is a standard FDK reconstruction.

**S**_**XCAT**_: Results obtained by a motion model fitted with the normalized input breathing traces from the XCAT simulations.

**S**_**IA**_: Results obtained by a motion model fitted with the normalized IA signal and its temporal derivative.

These three scenarios were assessed for both the regular and irregular breathing simulations.

## 4. Results

### 4.1 Extracted surrogate signal

**Figure 3.**
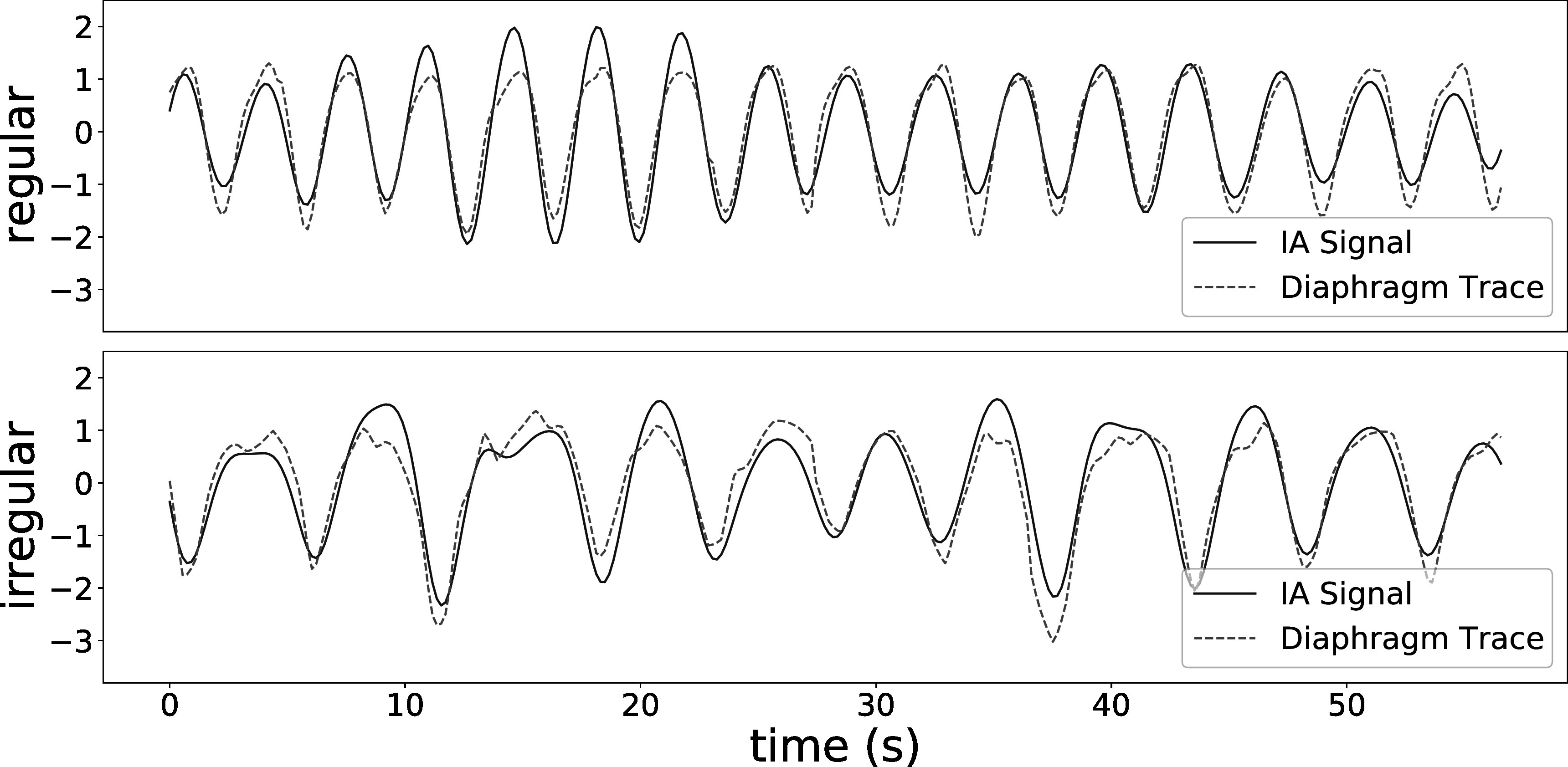
The normalized IA signals obtained from projection images (blue curves), overlapped on normalized diaphragm traces for the two 4DXCAT simulations (red curves).

**Figure 4.**
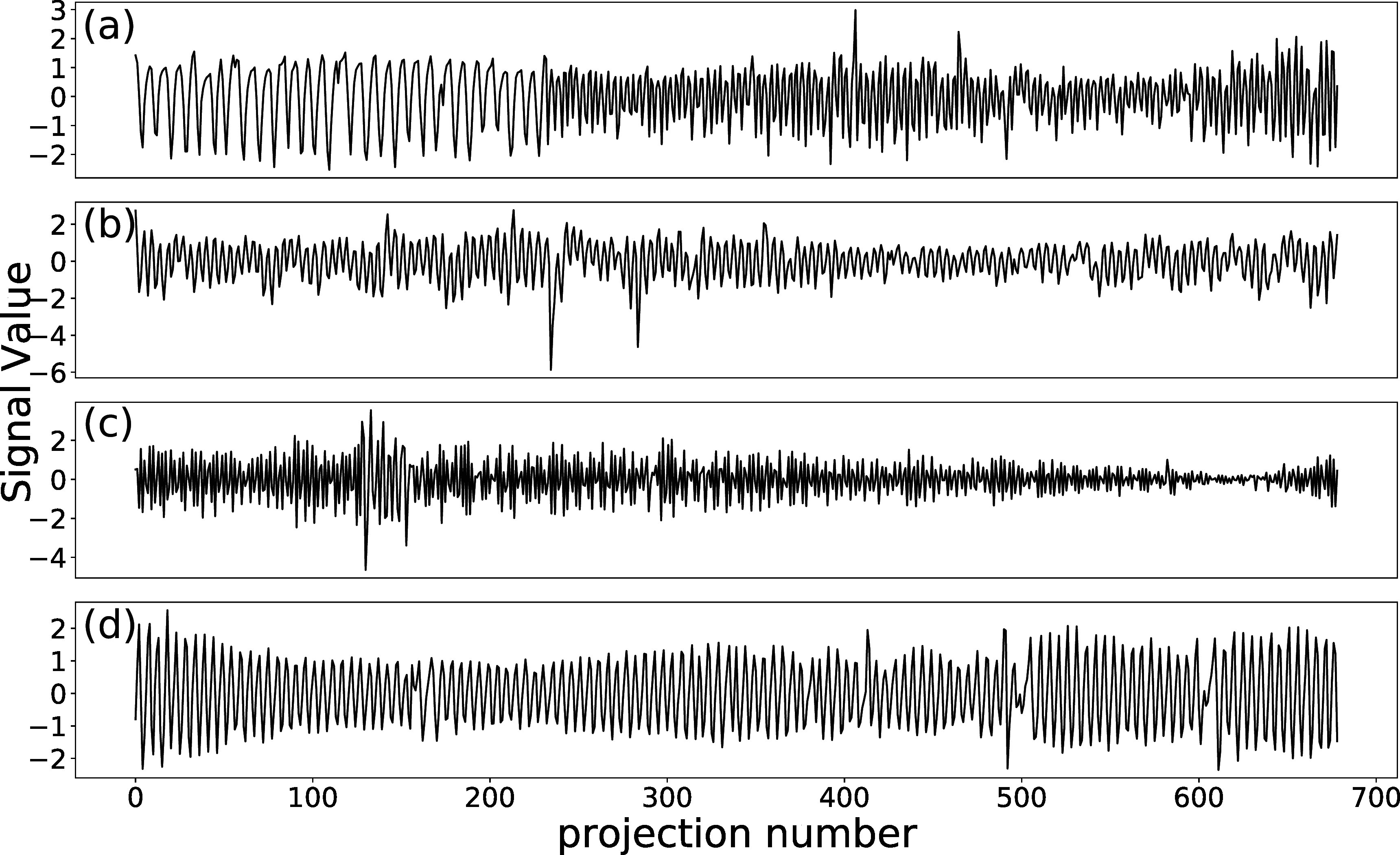
The IA signals obtained from projection images of four patients from the spare challenge.

**Figure 5.**
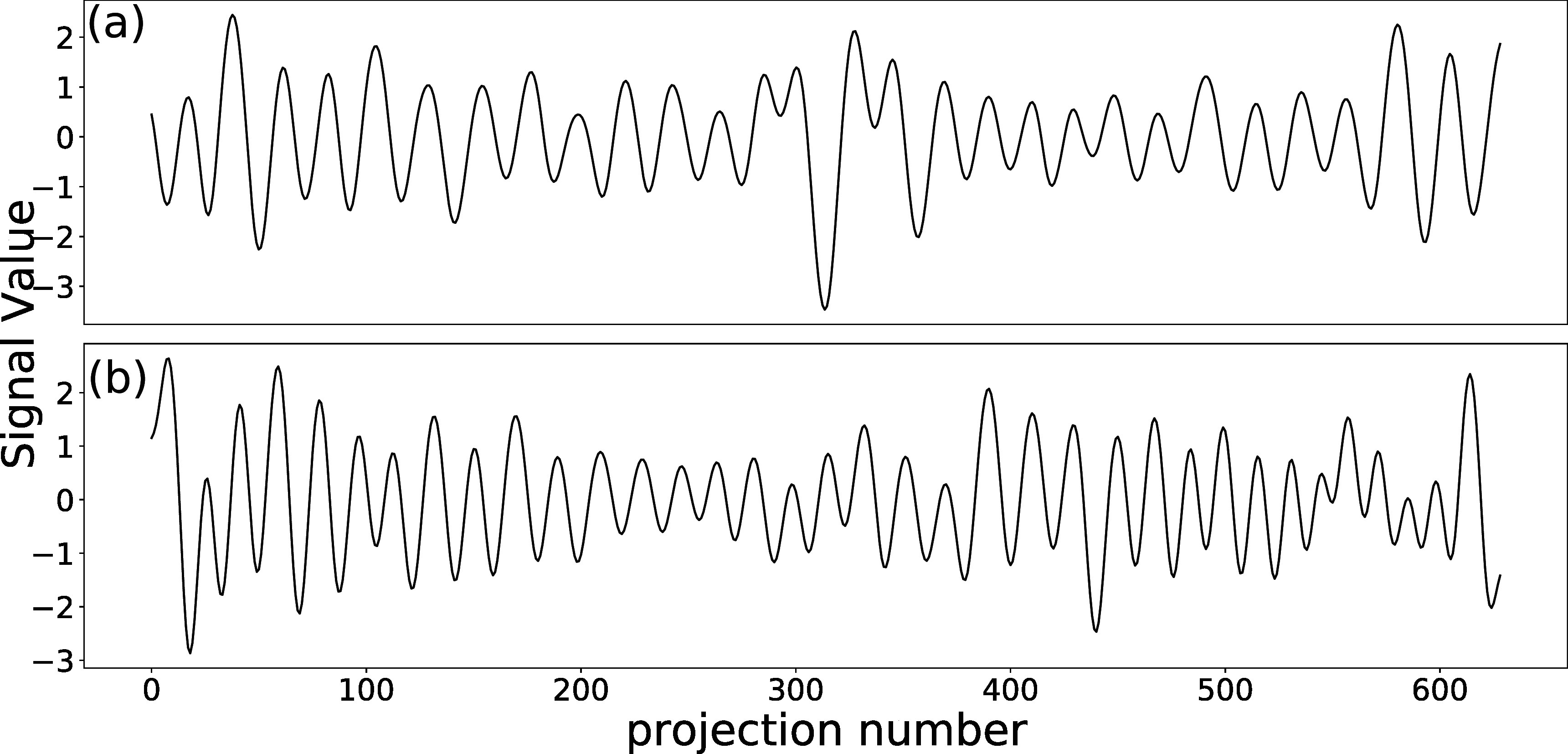
The IA signals obtained from projection images of two patients from ROSS-LC clinical trial.

Figure 3 displays the normalized IA signals extracted from projection images (blue curves), overlaid on normalized diaphragm traces (red curves) for the two 4DXCAT simulations. The IA signals are similar to the diaphragm signal over most timepoints (Pearson correlation coefficients: 0.936 and 0.920 for regular and irregular breathing respectively), although there are a few times where there are relatively large differences between the signals. Figure 4 shows the IA signals for the four patients from the Spare Challenge, respectively. For the real data there is no ground truth signal to compare to. It should be noted that the SPARE challenge provides datasets with subsampled sets of projection images from 8 min scans, such that the number of projection images are the same as would be available from 1 min scans (680 projection images). This is why the respiration seems to have a high frequency (although it is not clear why the frequency is lower for the first part of the first scan). Figure 5 shows the IA signals for the two patients from the ROSS-LC clinical trial, respectively. Here, the IA signals have more natural breathing frequencies because they were extracted from standard clinical scans.

### 4.2 Evaluation results for simulation data

Table 1 contains the results of the evaluation metrics for the three scenarios respectively, as described in section 3.2. The uncorrected results (**S**_**uncorr**_) show that there is substantial motion of the tumor and other anatomy during CBCT acquisition. When fitting the motion model with any type of surrogate signals (**S**_**XCAT**_/**S**_**IA**_), the accuracy of motion estimation and the quality of the reconstructed images have been improved. The DSC and E_center_ metrics show that tumor motion has been estimated accurately. E_DVF_ shows that motion everywhere else has also been estimated well. NRMSE and SSIM show that ${{\mathrm{I}}}_{0}^{\mathrm{est}}$ is more similar to ${{\mathrm{I}}}_{0}^{\mathrm{gt}}$ when a motion model is used.

**Table 1.** Evaluation metrics for regular and irregular breathing simulations (unit of E_center_ and E_DVF_: mm).

Simulation	Scenarios	E_D_	DSC	E_center_	NRMSE	SSIM
Regular breathing	**S**_**uncorr**_	2.38 ± 1.14	0.46 ± 0.23	8.32 ± 5.68	0.11	0.92
	**S**_**XCAT**_	1.34 ± 0.63	0.90 ± 0.05	0.93 ± 0.54	0.07	0.95
	**S**_**IA**_	1.58 ± 0.57	0.78 ± 0.11	2.41 ± 1.31	0.09	0.94
Irregular breathing	**S**_**uncorr**_	1.70 ± 0.68	0.63 ± 0.15	7.72 ± 3.70	0.10	0.94
	**S**_**XCAT**_	1.07 ± 0.46	0.92 ± 0.03	0.70 ± 0.31	0.08	0.96
	**S**_**IA**_	1.30 ± 0.56	0.83 ± 0.09	1.78 ± 1.04	0.09	0.95

**Figure 6.**
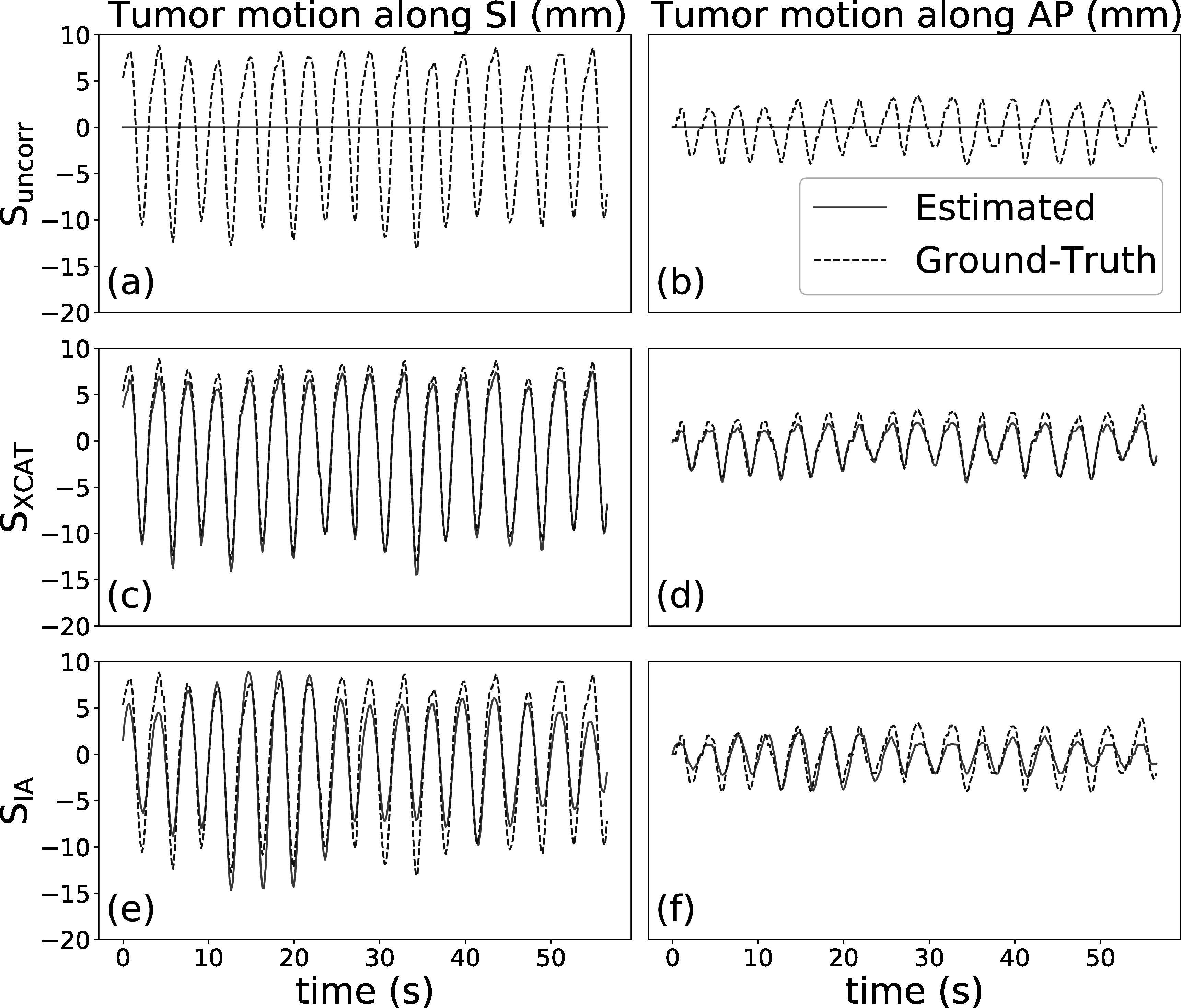
Estimated and ground-truth displacement of tumor centroid in SI (left) and AP (right) direction, for regular breathing simulation.

**Figure 7.**
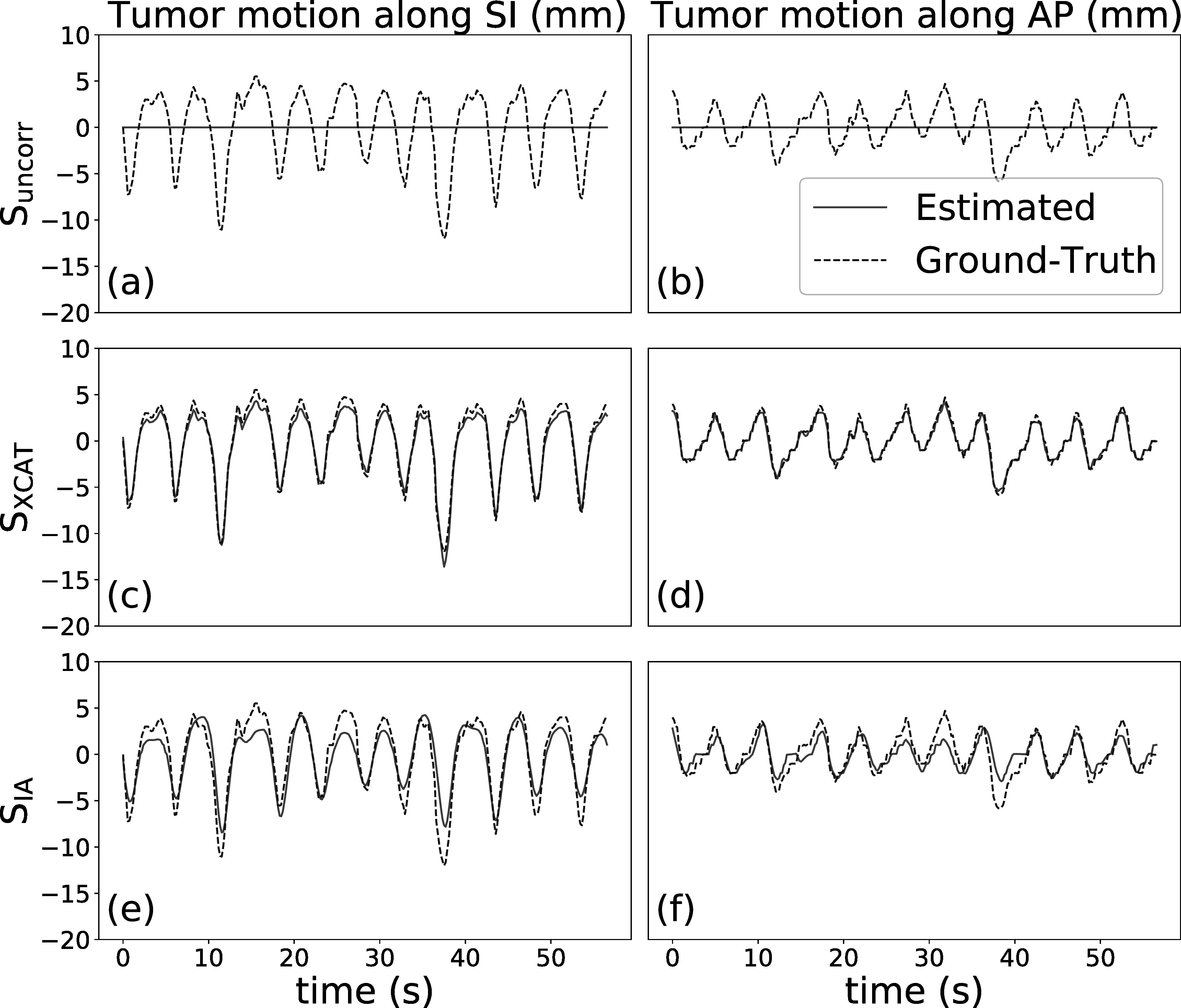
Estimated and ground-truth displacement of tumor centroid in SI (left) and AP (right) direction, for irregular breathing simulation.

**Figure 8.**
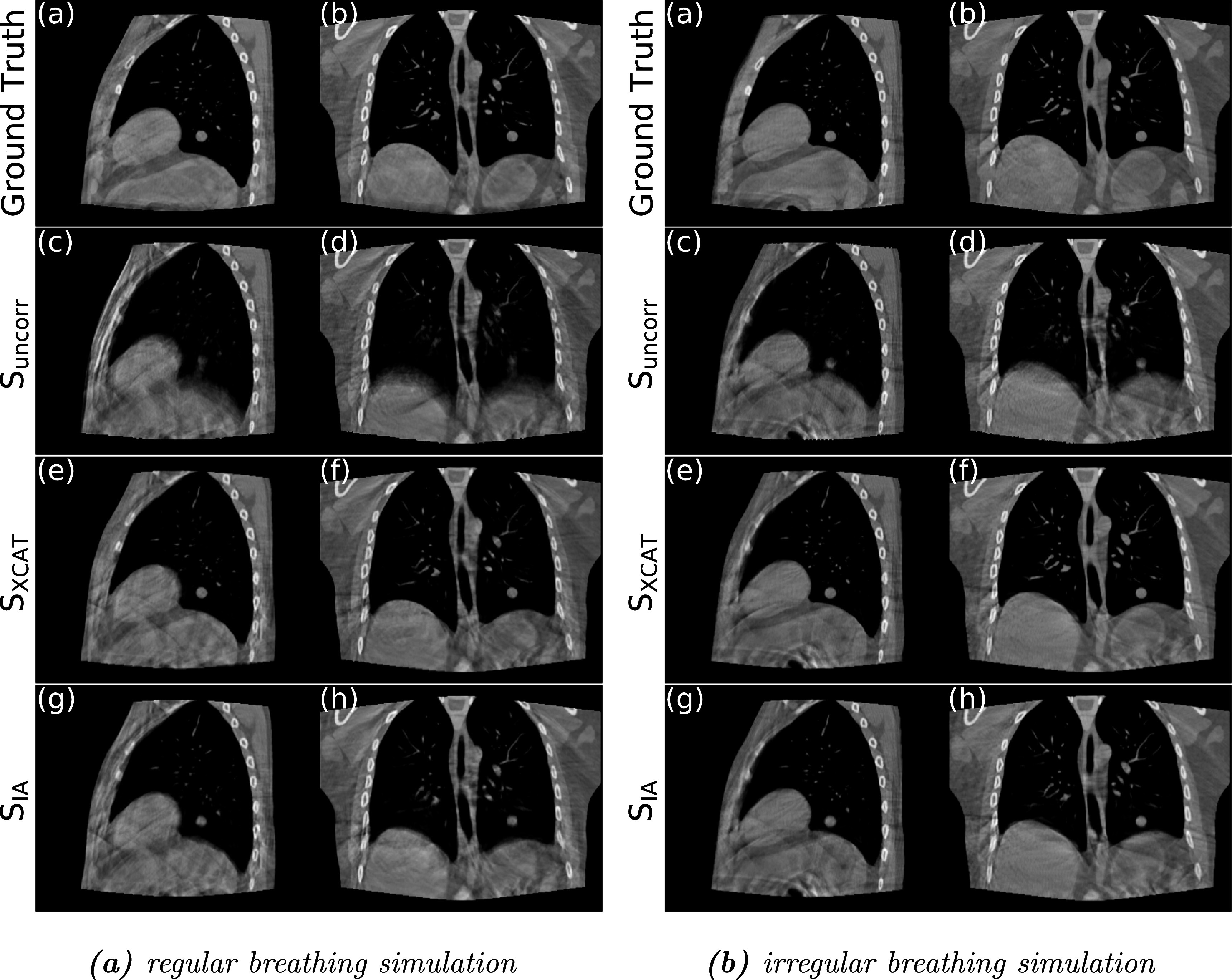
Sagittal and Coronal views of the ground-truth CBCT images, and the reconstruction under the three scenarios (**S**_**uncorr**_/**S**_**XCAT**_/**S**_**IA**_) for (a) regular and (b) irregular breathing simulation.

The regular and irregular breathing simulations are different in terms of magnitude and hysteresis of respiration. However, the improvement is observed for both simulations, showing that our method can model the intra- and inter-cycle variations seen in the simulations and can model the larger motion seen in the regular simulation as well as the smaller motion seen in the irregular simulation. For both simulations, the surrogate signals used as input to XCAT (**S**_**XCAT**_) give markedly better results than IA signals (**S**_**IA**_). Figures 6 and 7 demonstrate the displacement of tumor centroid at each frame in the SI (left column) and AP (right column) directions for the regular and irregular breathing simulation, respectively. These figures compare the three scenarios as explained section 3.2, **S**_**uncorr**_/**S**_**XCAT**_/**S**_**IA**_, in terms of their capability to track tumor motion. The red solid traces refer to the result without any motion (a)–(b) or obtained by the motion models (c)–(f), while the blue dashed traces refer to the ground-truth tumor centroid displacement. The Pearson correlation coefficients between estimated and ground-truth tumor displacement for **S**_**XCAT**_ are 0.997 [SI direction] and 0.976 [AP direction] for regular breathing, and 0.993 [SI direction] and 0.991 [AP direction] for irregular breathing. For **S**_**IA**_ they are 0.940 [SI direction] and 0.857 [AP direction] for regular breathing, and 0.930 [SI direction] and 0.845 [AP direction] for irregular breathing. For **S**_**uncorr**_ the correlation is always 0 since no motion is estimated. For both simulations, it can be seen that motion models fitted with the XCAT input traces can estimate the tumor motion with high accuracy, whereas the model fitted with the extracted IA signals is less accurate, although it still estimates most of the motion reasonably well. These results, together with those in table 1, suggest that the use of extracted signals which do not perfectly correspond to the internal motion can have a considerable impact on the accuracy of the motion models. Figures 8(a) and (b) display sagittal and coronal views of the ground-truth images, and the reconstruction images under the different scenarios listed in section 3.2. Here, ground-truth images refer to motion compensated FDK reconstruction using the known ground-truth motion.

From figures 8(a) and (b), it can be seen that the image quality of the standard FDK reconstruction (c), (d) is impacted by the motion, with the tumor, diaphragm, and other structures appearing blurry. This is more noticeable for the regular motion in figures 8(a) since the motion is larger for the regular simulation. When a motion compensated FDK is performed using the ground truth DVFs (a), (b) it can be seen that the motion is almost perfectly compensated for and all the blurring and other artifacts have been removed. The results from our method using the XCAT input traces (e), (f) are almost as good as when the ground-truth DVFs (a), (b) are used. The results from our method when using the extracted IA signals (i), (j) show a few more artifacts compare to the results using the XCAT input traces, with the tumor and part of the diaphragm slightly blurred (this is more noticeable for the regular simulation due to the larger magnitude of motion). However, even the results using the extracted surrogate signals show considerable improvement over the standard FDK reconstruction (c), (d). These visual assessment results are in good agreement with the quantitative results presented in table 1.

To demonstrate how different motion models change the anatomy temporally, two movies of animated CBCT images can be found in appendix A1 and A2 for regular and irregular breathing simulation respectively. Reference CBCT images are obtained by motion compensated FDK reconstruction and then animated by motion models using different surrogate signals (S_XCAT_/S_IA_). The red circles refer to the ground-truth tumor masks at each time-point, which are mostly consistent with the tumor boundary seen in the animated CBCT images.

### 4.3 Evaluation results for real patient data

**Figure 9.**
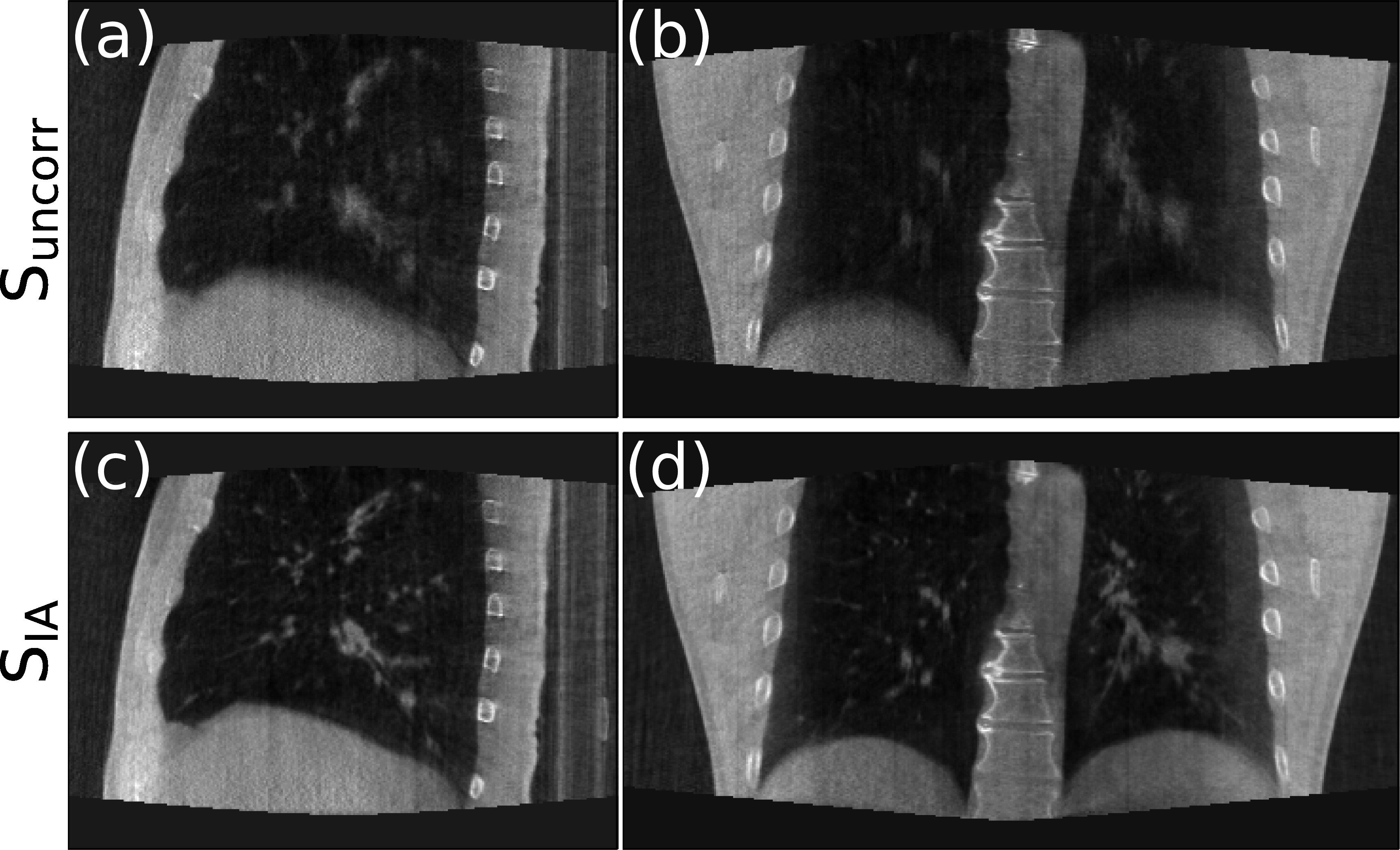
Standard FDK reconstruction (top) and motion compensated reconstruction with our method using IA signal for patient 1 in SPARE challenge.

**Figure 10.**
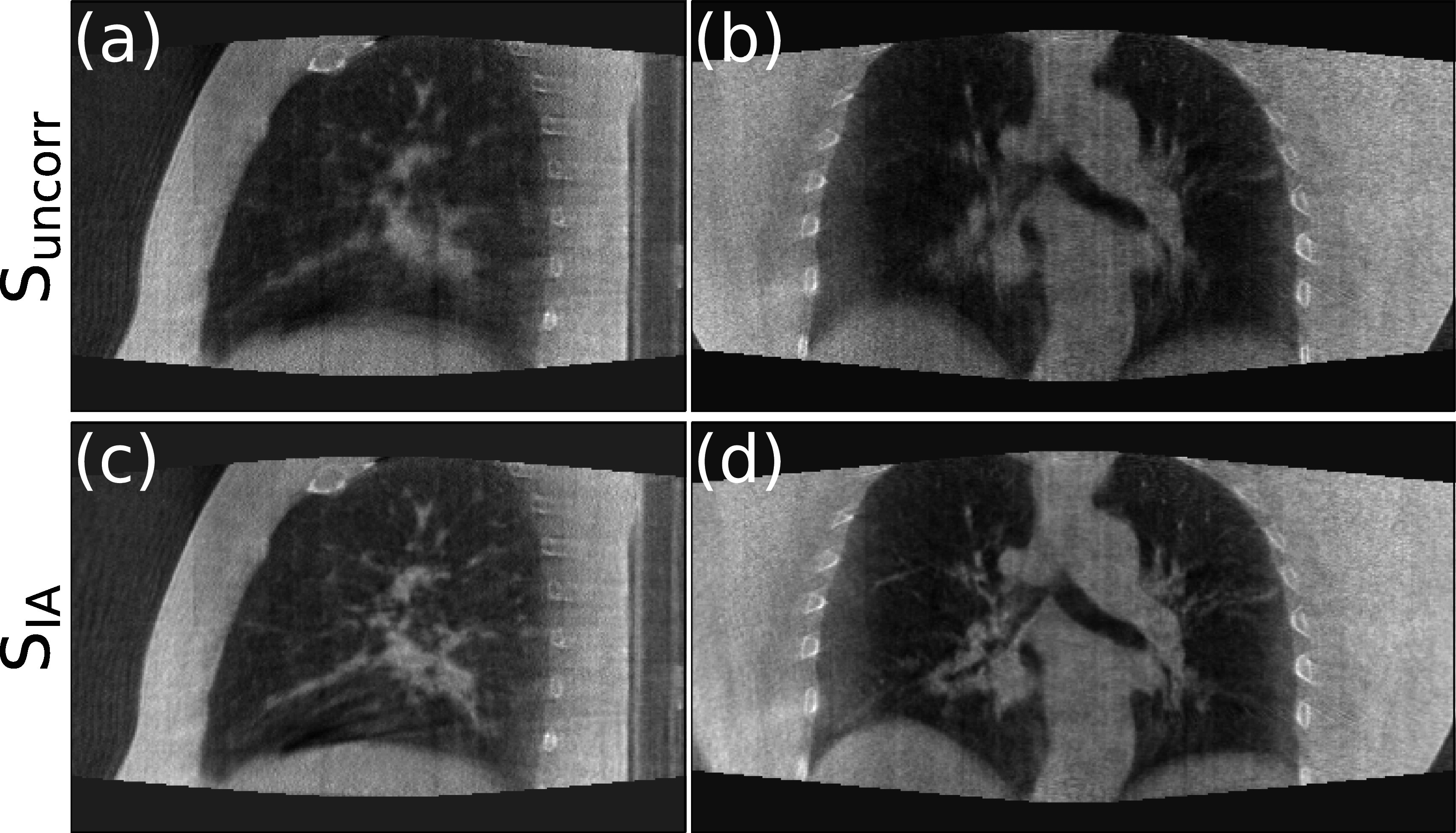
Standard FDK reconstruction (top) and motion compensated reconstruction with our method using IA signal for patient 2 in SPARE challenge.

Figures 9 and 10 show the sagittal (left column) and coronal (right column) views of standard FDK reconstructions (a)–(b) and motion compensated reconstructions using our method (c)–(d) for the two patients from the SPARE challenge dataset with the most motion. Similar to the observation for the simulated data, clearer lung tissue details and sharper diaphragm edges can be observed in the reconstructed CBCT after applying our method for both patients. Similar results of two more patients in SPARE Challenge dataset can be found in appendix A3 and A4. These two patients exhibit less motion, so there are less motion artifacts in the original CBCTs, but there are stil some noticeable improvements in the motion compensated images produced by our method.

Benchmark results in SPARE Challenge did not include CBCT images at each projection time-point. To compare with the results from the SPARE Challenge (Data S6 in Shieh *et al* (2019)), we have created a ‘synthetic’ 4DCBCT for the first patient from SPARE challenge, which can be found in appendix A5. This was generated by animating the motion compensated reconstruction with our motion model and the average values of the surrogate signals of each phase bin. The quality of the synthetic 4DCBCT using our method was comparable to the best results from the SPARE challenge, but it should be emphasized that our method also has the ability to provide frame-by-frame CBCT images over all projections, and thus can estimate CBCTs exhibiting breath-to-breath variation.

**Figure 11.**
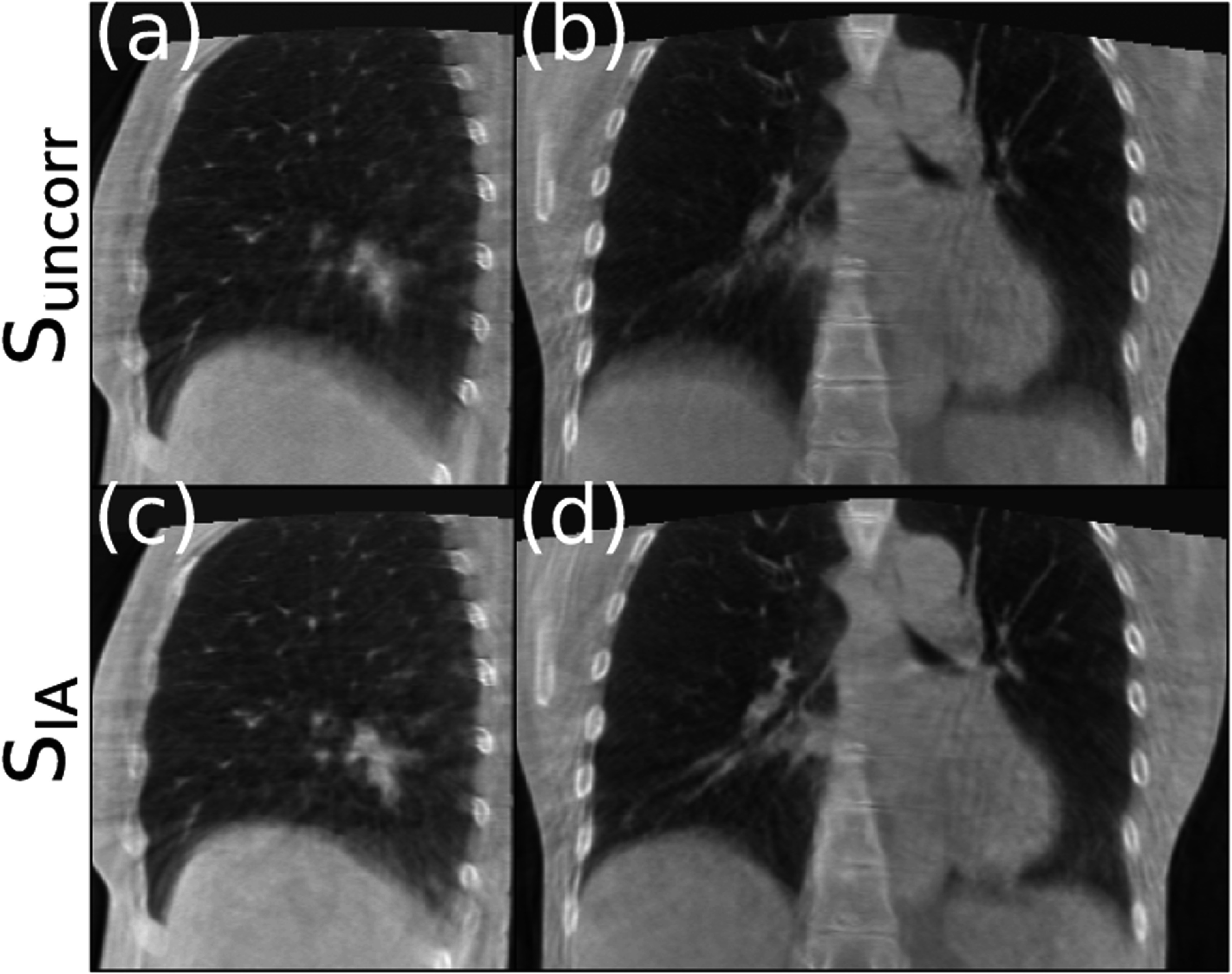
Standard FDK reconstruction (top) and motion compensated reconstruction with our method using IA signals (bottom) for first patient of clinical trial.

**Figure 12.**
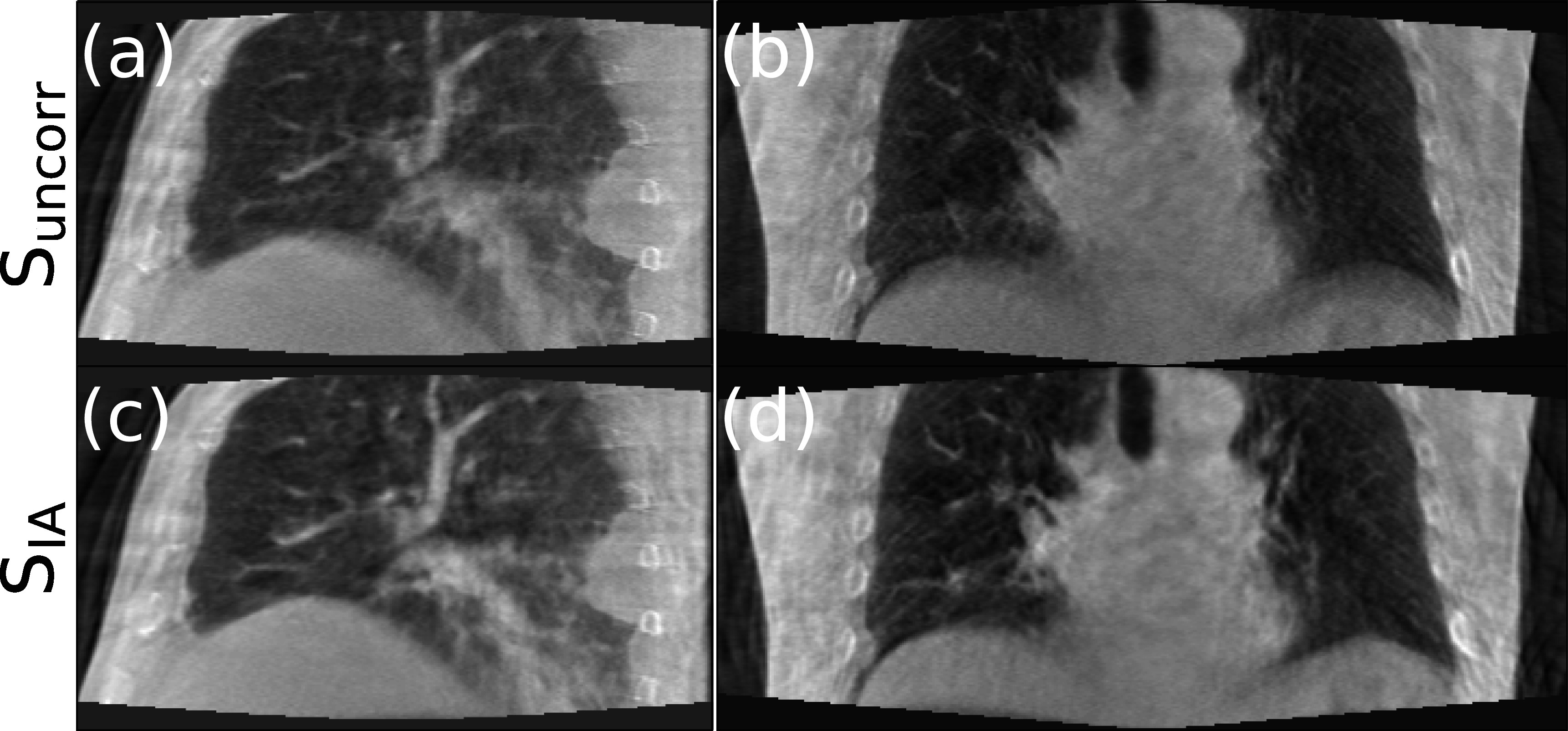
Standard FDK reconstruction (top) and motion compensated reconstruction with our method using IA signals (bottom) for second patient of clinical trial.

Similarly, figures 11 and 12 display sagittal (left column) and coronal (right column) views of the reconstructions for the two patients from the ROSS-LC clinical trial. It can be seen in both figures that the edge of airways and diaphragm look sharper after applying our method, similar to the results for the SPARE challenge datasets and simulated data.

Appendix A6 and A7 display movies of the animated CBCT images at each time-point for the two scans in ROSS-LC clinical trial, estimated by our method. The movies show that the overall motion estimated by our method is generally plausible. However, it is evident that the sliding motion between the tumor and the ribs for the first patient has not been perfectly modeled. This is expected due to the use of the B-spline FFD transformation model and will be addressed in future work.

We showed all the results of real patients to two experienced radiation oncologists who agreed that in all cases the motion compensated images contained less motion artifacts than the standard FDK reconstructions, and this could facilitate more accurate monitoring and delineation of the tumor and other organs in the CBCT scans.

## 5. Discussion and conclusion

The major contribution of this work is obtaining a motion-free reconstruction and frame-by-frame DVFs just from unsorted projection data of a standard clinical 3DCBCT scan. Performance of our method has been validated on simulated and real data, showing promising results. It should be emphasized that the aim of our study is not to improve 4DCBCT. Rather, the aim of our study is to estimate the motion for every projection from a standard 3DCBCT scan and use the estimated motion to reconstruct a single 3D motion compensated image. Unlike 4DCBCT, and methods that attempt to improve 4DCBCT image quality, our method can model breath-to-breath variation in the motion and just requires projection data from a standard 3DCBCT scan. The motion compensated image from our method can be animated using the estimated motion to produce CBCT images corresponding to all of the projections, and which exhibit breath-to-breath variability. The general framework (McClelland *et al* 2017) in this study makes it possible to fit the motion model directly on CBCT projections. When using a traditional approach for fitting surrogate-driven motion models (McClelland *et al* 2013), image registration needs to be performed separately for each time-point to obtain the DVFs prior to fitting the motion model, but this requires volumetric images. In comparison, our framework integrates image registration and motion model fitting into a unified process so that the surrogate-driven motion model can be fitted directly to the projection data.

When applying our method, there are several technical points that need consideration. The choice of similarity measure is critical. Sum-of-Squared-Difference or similar measures (e.g. mean-absolute-difference) may be suitable for simulated data where both the measured projection images and model estimated projection images are produced by OpenRTK and so have similar intensities. However, there is an intrinsic difference of pixel values between the measured and model estimated projections in real patient data, due to the more complicated physical process such as beam hardening and scattering, etc. Therefore, LNCC was used as the similarity measure, which assumes a linear relationship between the intensities in the measured and estimated projections, but allows this relationship to vary across the image.

The number of surrogate signals is another essential factor. For data like the irregular breathing simulation, the motion of chest skin surface and diaphragm is hysteretic, i.e. out-of-phase with each other. Fitting the motion model with just one surrogate signal can only recover the motion in the dominant direction, e.g. the SI direction. At least two signals are required to model the out-of-phase hysteretic motion. While increasing the number of surrogate signals can strengthen the ability to model more complex or variable motion, the danger of overfitting and thus need for larger dataset should be considered with caution. Since it has been reported that respiration motion can be modeled well with two signals/components (Tran *et al* 2020) we used two surrogate signals in this study.

It is also noticeable that the method used to extract the surrogate signals can have a considerable influence on the results. We also investigated the more well-known Amsterdam Shroud (AS) method (Zijp *et al* 2004) as well as the IA method, but found it gave unsatisfactory results for all the real scans except the first ROSS-LC clinical trail scan. We speculate that this could be due to the SPARE challenge datasets being sub-sampled from a longer scan, and the small FOV in the second ROSS-LC trial scan meaning the diaphragm was not present in all projections. For the XCAT simulations, the IA method produces signals that match the input diaphragm signal reasonably well. However, the models built using the extracted IA signals have noticeably worse results than the model built with the XCAT input signals, indicating that using the extracted signals can negatively impact the model’s accuracy even when the signals appear plausible. More advanced surrogate extraction methods or external devices may generate more suitable surrogate signals in some cases and give better results, but in general it is still a challenge to reliably acquire good signals that have a strong and consistent relationship with the internal motion. An alternative approach is to develop models that do not rely on good surrogate signals as input, and we are currently working on such models.

Despite the issues with the extracted surrogate signals, it should be noted that our method has produced very promising looking results on six real datasets. There have been other studies that attempt to produce similar results as we have in this paper, i.e. DVFs for every projection, that can include breath-to-breath variability, and a motion-free reconstruction, (Liu *et al* 2015, Jailin *et al* 2021, Zhang *et al* 2023). However, Liu *et al* (2015) only applied their method to simulated data from a simplified 2D simulation (i.e. the anatomy and motion was only 2D). The method in (Zhang *et al* 2023) can only be applied to low resolution data due to GPU memory constraints, and has only been demonstrated on simulated data. Jailin *et al* (2021) did apply their method to real data, but only demonstrated it on a single scan, and it required very long computation times (∼30 h). As far as we are aware this is the first time such a method has been applied to multiple real CBCT datasets. We believe our method is less complicated than those presented in (Liu *et al* 2015, Jailin *et al* 2021, Zhang *et al* 2023). The runtime for our method ranged from 30 to 120 min for the real CBCT scans on an Intel Core i7-10700K CPU. We acknowledge that this is still too long for clinical use, but in the future our method will be implemented to run on a GPU and the code will be further optimised to reduce runtime, which we expect will enable clinically usable runtimes of a few minutes.

Another limitation of our method is that we currently require non-truncated data, as the missing anatomy in the reconstruction contributes to the measured projection but not to the estimated projections, causing inherent mismatch between the measured and estimated projections and thus interfering with motion estimation. More advanced reconstruction algorithms, such as iterative reconstruction algorithms, will be investigated to overcome the truncation issue. Another potential solution is to use an existing image that contains all of the anatomy, e.g. from the planning CT, as the reference state image, *I*_0_, instead of using the motion compensated CBCT. As well as overcoming the issue with truncated data this can provide a synthetic CT and updated structure delineations by deforming the planning CT and structures, facilitating dose calculations. However, this approach could struggle if there are substantial anatomical changes between the reference image and the daily anatomy.

Ourmethod has great potential for future clinical applications as it can provide both a high-quality motion compensated CBCT image, and accurate estimates of the respiratory motion, including intra- and inter-cycle variations, from nothing other than projection data of a standard 3DCBCT scan. This means it can provide up-to-date estimates of the image and motion of the day on standard linacs, facilitating future innovations in adaptive treatments and outcome studies by providing up-to-date targets and OARs delineation, and more accurate estimates of the delivered dose.

## Figures and Tables

**Figure A1.**
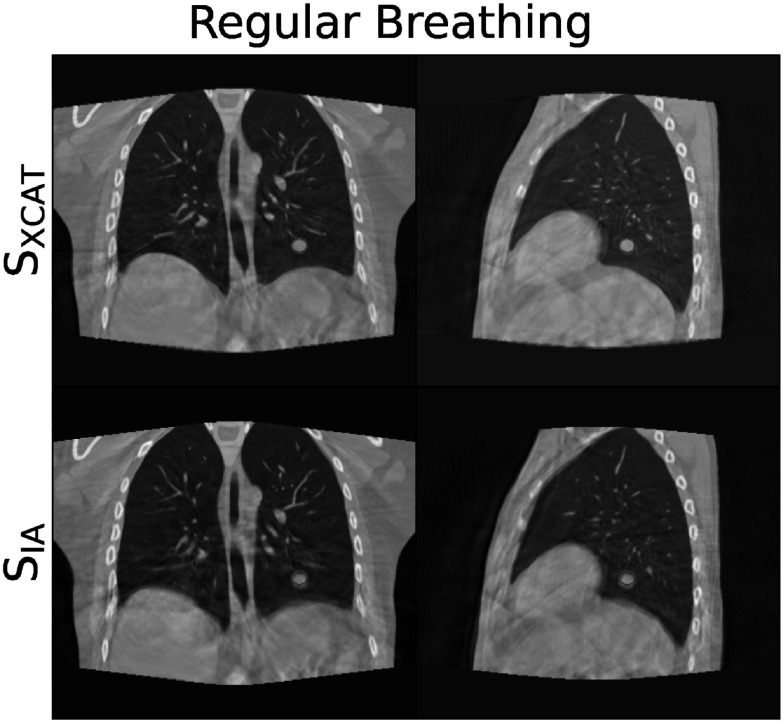
Static image of supplementary movie **A1**. Movie can be found in online version.

**Figure A2.**
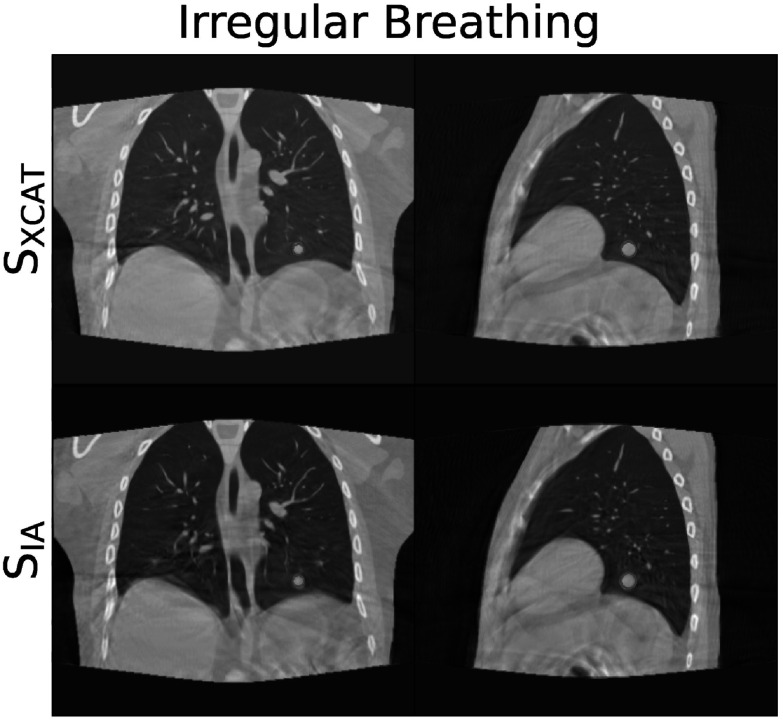
Static image of supplementary movie **A2**. Movie can be found in online version.

**Figure A3.**
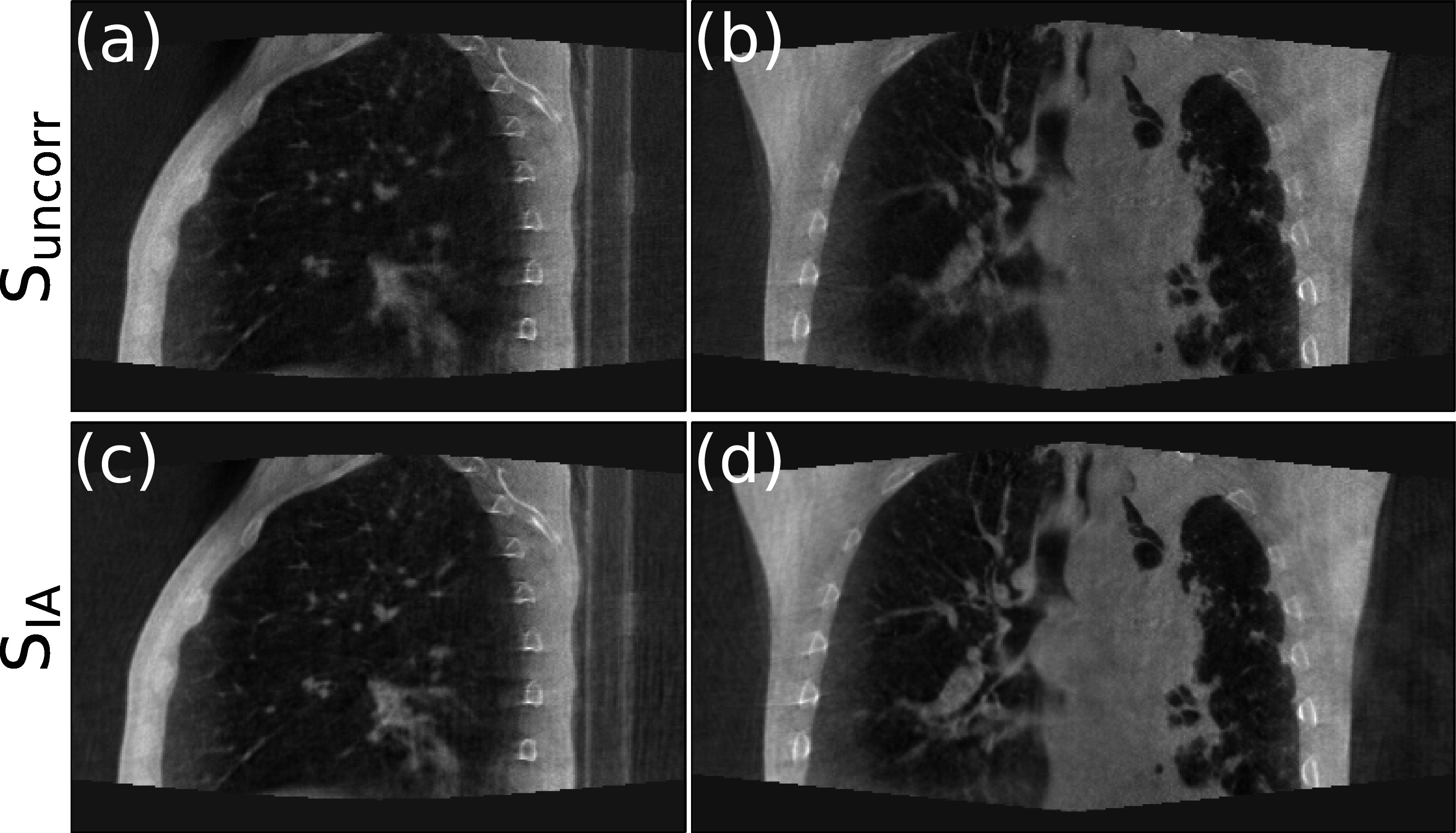
Supplementary image **A3**.

**Figure A4.**
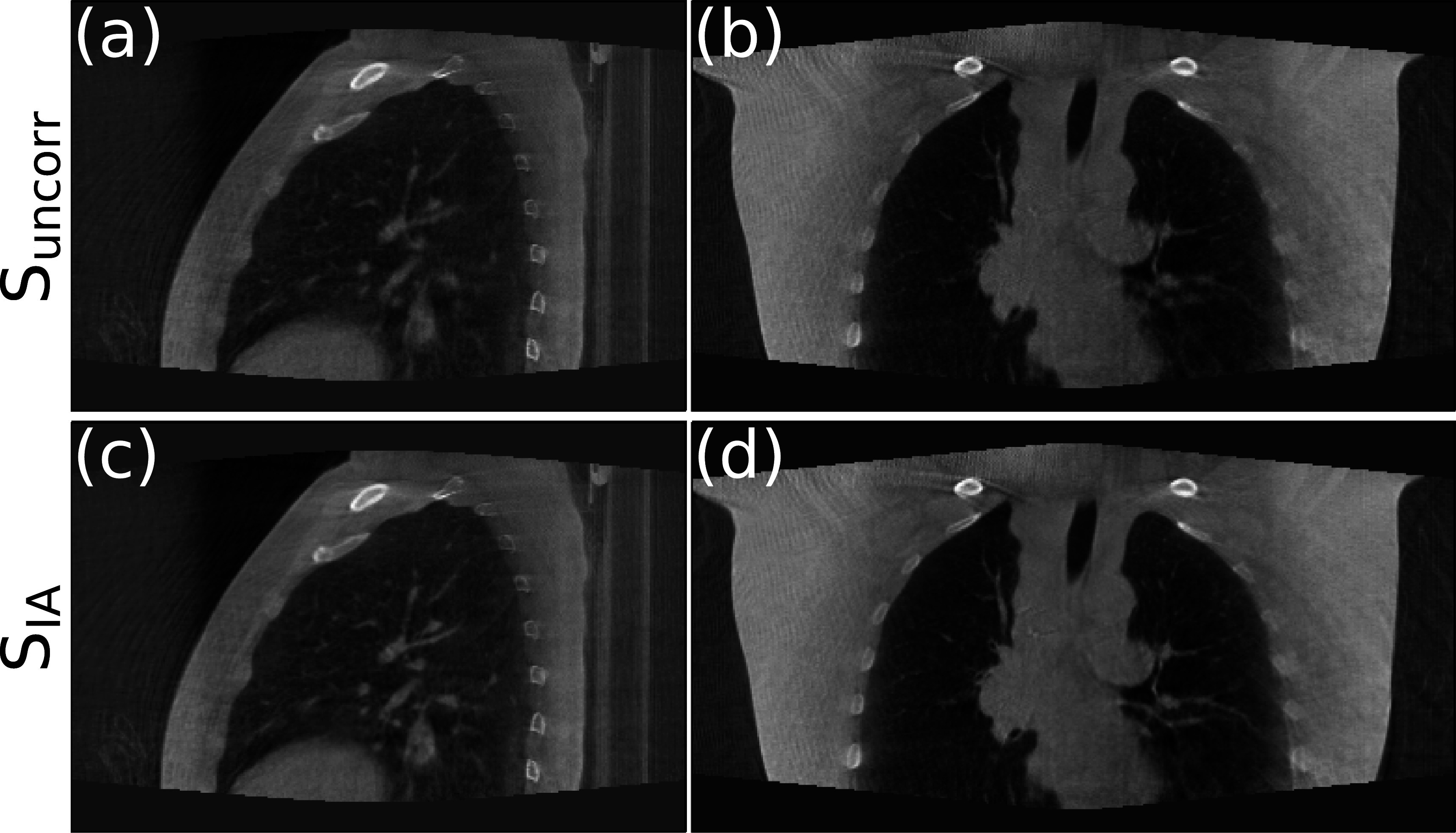
Supplementary image **A4**.

**Figure A5.**
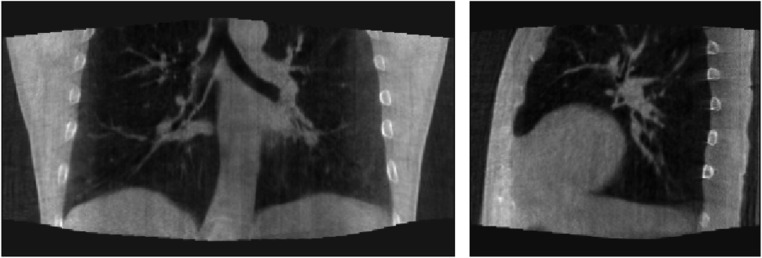
Static image of supplementary movie **A5**. Movie can be found in online version.

**Figure A6.**
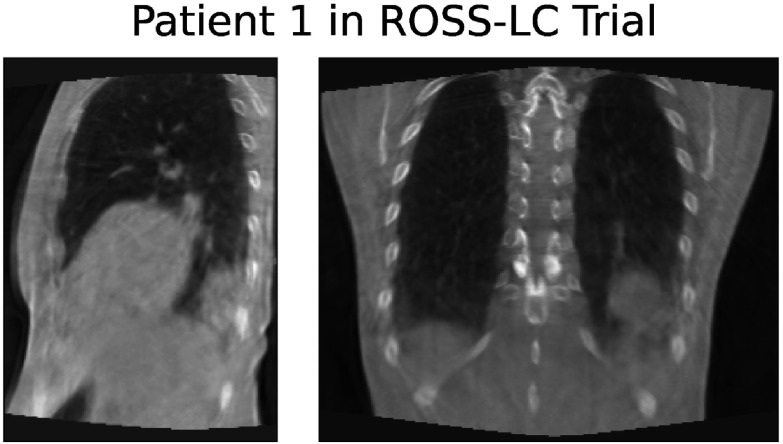
Static image of supplementary movie **A6**. Movie can be found in online version.

**Figure A7.**
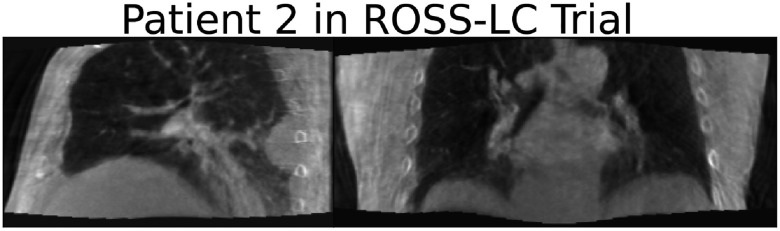
Static image of supplementary movie **A7**. Movie can be found in online version.

## Data Availability

We are happy to share all our simulated data upon request. The clinical data cannot be made publicly available upon publication because they are owned by a third party and the terms of use prevent public distribution. The data that support the findings of this study are available upon reasonable request from the authors.

## Appendix

**A1**: Animation of motion compensated CBCT images using motion models from different surrogate signals (S_XCAT_/S_IA_) for regular breathing simulation. Ground-truth tumor masks (red circles) are overlapped on CBCT images.

**A2**: Animation of motion compensated CBCT images using motion models from different surrogate signals (S_XCAT_/S_IA_) for irregular breathing simulation. Ground-truth tumor masks (red circles) are overlapped on CBCT images.

**A3**: Standard FDK reconstruction (top) and motion compensated reconstruction with our method using IA signal for patient 3 in SPARE challenge.

**A4**: Standard FDK reconstruction (top) and motion compensated reconstruction with our method using IA signal for patient 4 in SPARE challenge.

**A5**: Synthetic 4DCBCT of the first patient from SPARE challenge using average value of IA signals within each phase. Bcnd in Data S6 in Shieh *et al* (2019).

**A6**: Movies of CBCT images estimated by our method with IA signals at each time-point for patient 1 in ROSS-LC clinical trial.

**A7**: Movies of CBCT images estimated by our method with IA signals at each time-point for patient 2 in ROSS-LC clinical trial.

## References

[pmbad1546bib1] Brown S, Banfill K, Aznar M C, Whitehurst P, Faivre Finn C (2019). The evolving role of radiotherapy in non-small cell lung
cancer. Br. J. Radiol..

[pmbad1546bib2] Chee G, O’Connell D, Yang Y, Singhrao K, Low D, Lewis J (2019). Mcsart: an iterative model-based, motion-compensated sart
algorithm for CBCT reconstruction. Phys. Med. Biol..

[pmbad1546bib3] Chen G H, Tang J, Leng S (2008). Prior image constrained compressed sensing (PICCS): a method to
accurately reconstruct dynamic ct images from highly undersampled projection
data sets. Med. Phys..

[pmbad1546bib4] Cole A, Veiga C, Johnson U, D’Souza D, Lalli N, McClelland J (2018). Toward adaptive radiotherapy for lung patients: feasibility study
on deforming planning CT to CBCT to assess the impact of anatomical changes
on dosimetry. Phys. Med. Biol..

[pmbad1546bib5] De Los Santos J (2013). Image guided radiation therapy (igrt) technologies for radiation
therapy localization and delivery. Int. J. Radiat. Oncol. Biol. Phys..

[pmbad1546bib6] den Otter L A (2020). Investigation of inter-fraction target motion variations in the
context of pencil beam scanned proton therapy in non-small cell lung cancer
patients. Med. Phys..

[pmbad1546bib7] Dhont J (2018). The long-and short-term variability of breathing induced tumor
motion in lung and liver over the course of a radiotherapy
treatment. Radiother. Oncol..

[pmbad1546bib8] Dong Z, Yu S, Szmul A, Wang J, Qi J, Wu H, Li J, Lu Z, Zhang Y (2023). Simulation of a new respiratory phase sorting method for
4D-imaging using optical surface information towards precision
radiotherapy. Comput. Biol. Med..

[pmbad1546bib9] Eiben B, Bertholet J, Menten M J, Nill S, Oelfke U, McClelland J R (2020). Consistent and invertible deformation vector fields for a
breathing anthropomorphic phantom: a post-processing framework for the XCAT
phantom. Phys. Med. Biol..

[pmbad1546bib10] Feldkamp L A, Davis L C, Kress J W (1984). Practical cone-beam algorithm. J. Opt. Soc. Am. A.

[pmbad1546bib11] Guo M (2019). Reconstruction of a high-quality volumetric image and a
respiratory motion model from patient CBCT projections. Med. Phys..

[pmbad1546bib12] Huang Y, Thielemans K, McClelland J R (2023). Surrogate-driven motion model for motion compensated cone-beam ct
reconstruction using unsorted projection data.

[pmbad1546bib13] Hurwitz M, Williams C L, Mishra P, Rottmann J, Dhou S, Wagar M, Mannarino E G, Mak R H, Lewis J H (2014). Generation of fluoroscopic 3D images with a respiratory motion
model based on an external surrogate signal. Phys. Med. Biol..

[pmbad1546bib14] Jailin C, Roux S, Sarrut D, Rit S (2021). Projection-based dynamic tomography. Phys. Med. Biol..

[pmbad1546bib15] Jia X, Lou Y, Li R, Song W Y, Jiang S B (2010). Gpu-based fast cone beam ct reconstruction from undersampled and
noisy projection data via total variation. Med. Phys..

[pmbad1546bib16] Jiang Z, Chen Y, Zhang Y, Ge Y, Yin F F, Ren L (2019). Augmentation of CBCT reconstructed from under-sampled projections
using deep learning. IEEE Trans. Med. Imaging.

[pmbad1546bib17] Kavanagh A, Evans P M, Hansen V N, Webb S (2009). Obtaining breathing patterns from any sequential thoracic x-ray
image set. Phys. Med. Biol..

[pmbad1546bib18] Leng S, Zambelli J, Tolakanahalli R, Nett B, Munro P, Star-Lack J, Paliwal B, Chen G H (2008). Streaking artifacts reduction in four-dimensional cone-beam
computed tomography. Med. Phys..

[pmbad1546bib19] Liu F, Hu Y, Zhang Q, Kincaid R, Goodman K, Mageras G (2012). Evaluation of deformable image registration and a motion model in
CT images with limited features. Phys. Med. Biol..

[pmbad1546bib20] Liu J, Zhang X, Zhang X, Zhao H, Gao Y, Thomas D, Low D A, Gao H (2015). 5d respiratory motion model based image reconstruction algorithm
for 4D cone-beam computed tomography. Inverse Prob..

[pmbad1546bib21] Low D A, Parikh P J, Lu W, Dempsey J F, Wahab S H, Hubenschmidt J P, Nystrom M M, Handoko M, Bradley J D (2005). Novel breathing motion model for radiotherapy. Int. J. Radiat. Oncol. Biol. Phys..

[pmbad1546bib22] Manber R, Thielemans K, Hutton B F, Wan S, McClelland J, Barnes A, Arridge S, Ourselin S, Atkinson D (2016). Joint pet-mr respiratory motion models for clinical pet motion
correction. Phys. Med. Biol..

[pmbad1546bib23] McClelland J R, Hawkes D J, Schaeffter T, King A P (2013). Respiratory motion models: a review. Med. Image Anal..

[pmbad1546bib24] McClelland J R (2017). A generalized framework unifying image registration and
respiratory motion models and incorporating image reconstruction, for
partial image data or full images. Phys. Med. Biol..

[pmbad1546bib25] Mory C (2014). Cardiac c-arm computed tomography using a 3D+ time roi
reconstruction method with spatial and temporal
regularization. Med. Phys..

[pmbad1546bib26] Mory C, Janssens G, Rit S (2016). Motion-aware temporal regularization for improved 4D cone-beam
computed tomography. Phys. Med. Biol..

[pmbad1546bib27] Nøttrup T J, Korreman S S, Pedersen A N, Aarup L R, Nyström H, Olsen M, Specht L (2007). Intra-and interfraction breathing variations during curative
radiotherapy for lung cancer. Radiother. Oncol..

[pmbad1546bib28] Pirzkall A, Lohr F, Höss A, Wannenmacher M, Debus J, Carol M (2000). Comparison of intensity-modulated radiotherapy with conventional
conformal radiotherapy for complex-shaped tumors. Int. J. Radiat. Oncol. Biol. Phys..

[pmbad1546bib29] Price G J, Faivre-Finn C, Stratford J, Chauhan S, Bewley M, Clarke L, Johnson C N, Moore C J (2018). Results from a clinical trial evaluating the efficacy of
real-time body surface visual feedback in reducing patient motion during
lung cancer radiotherapy. Acta Oncol..

[pmbad1546bib30] Rit S, Sarrut D, Desbat L (2009). Comparison of analytic and algebraic methods for
motion-compensated cone-beam ct reconstruction of the thorax. IEEE Trans. Med. Imaging.

[pmbad1546bib31] Rit S, Wolthaus J W, van Herk M, Sonke J J (2009). On-the-fly motion-compensated cone-beam ct using an a priori
model of the respiratory motion. Med. Phys..

[pmbad1546bib32] Rit S, Oliva M V, Brousmiche S, Labarbe R, Sarrut D, Sharp G C (2014). The reconstruction toolkit (rtk), an open-source cone-beam ct
reconstruction toolkit based on the insight toolkit (itk). J. Phys. Conf. Ser..

[pmbad1546bib33] Segars W P, Sturgeon G, Mendonca S, Grimes J, Tsui B M (2010). 4D XCAT phantom for multimodality imaging
research. Med. Phys..

[pmbad1546bib34] Shieh C C (2019). Spare: sparse-view reconstruction challenge for 4D cone-beam ct
from a 1 min scan. Med. Phys..

[pmbad1546bib35] Sonke J J, Zijp L, Remeijer P, Van Herk M (2005). Respiratory correlated cone beam ct. Med. Phys..

[pmbad1546bib36] Sweeney R A, Seubert B, Stark S, Homann V, Müller G, Flentje M, Guckenberger M (2012). Accuracy and inter-observer variability of 3d versus 4D cone-beam
ct based image-guidance in sbrt for lung tumors. Radiat. Oncol..

[pmbad1546bib37] Thengumpallil S, Smith K, Monnin P, Bourhis J, Bochud F, Moeckli R (2016). Difference in performance between 3d and 4D CBCT for lung
imaging: a dose and image quality analysis. J. Appl. Clin. Med. Phys..

[pmbad1546bib38] Tran E H, Eiben B, Wetscherek A, Oelfke U, Meedt G, Hawkes D J, McClelland J R (2020). Evaluation of mri-derived surrogate signals to model respiratory
motion. Biomed. Phys. Eng. Express.

[pmbad1546bib39] Tran E H (2022). Surrogate-driven respiratory motion models for MRI-guided lung
radiotherapy treatments. PhD Thesis.

[pmbad1546bib40] Wang J, Gu X (2013). Simultaneous motion estimation and image reconstruction (smeir)
for 4D cone-beam CT. Med. Phys..

[pmbad1546bib41] Yang P, Ge X, Tsui T, Liang X, Xie Y, Hu Z, Niu T (2022). Four-dimensional cone beam ct imaging using a single routine scan
via deep learning. IEEE Trans. Med. Imaging.

[pmbad1546bib42] Zhang Z, Liu J, Yang D, Kamilov U S, Hugo G D (2023). Deep learning-based motion compensation for four-dimensional
cone-beam computed tomography (4D-CBCT) reconstruction. Med. Phys..

[pmbad1546bib43] Zhang Y, Shao H C, Pan T, Mengke T (2023). Dynamic cone-beam ct reconstruction using spatial and temporal
implicit neural representation learning (stinr). Phys. Med. Biol..

[pmbad1546bib44] Zhao T, Lu W, Yang D, Mutic S, Noel C E, Parikh P J, Bradley J D, Low D A (2009). Characterization of free breathing patterns with 5D lung motion
model. Med. Phys..

[pmbad1546bib45] Zhi S, Kachelrieß M, Mou X (2021). Spatiotemporal structure-aware dictionary learning-based 4D CBCT
reconstruction. Med. Phys..

[pmbad1546bib46] Zijp L, Sonke J J, van Herk M (2004). Extraction of the respiratory signal from sequential thorax
cone-beam x-ray images.

